# Regulatory Phosphorylation of Ikaros by Bruton's Tyrosine Kinase

**DOI:** 10.1371/journal.pone.0071302

**Published:** 2013-08-19

**Authors:** Hong Ma, Sanjive Qazi, Zahide Ozer, Jian Zhang, Rita Ishkhanian, Fatih M. Uckun

**Affiliations:** 1 Systems Immunobiology Laboratory, Children's Center for Cancer and Blood Diseases, Children's Hospital Los Angeles, Los Angeles, California, United States of America; 2 Department of Biology and Bioinformatics Program, Gustavus Adolphus College, St. Peter, Minnesota, United States of America; 3 Molecular Oncology Program, Parker Hughes Institute, St. Paul, Minnesota, United States of America; 4 Medicinal Bioinformatics Center, Shanghai Jiatong University, Shanghai, China; 5 Department of Pediatrics, University of Southern California Keck School of Medicine, Los Angeles, California, United States of America; Westmead Millennium Institute, University of Sydney, Australia

## Abstract

Diminished Ikaros function has been implicated in the pathogenesis of acute lymphoblastic leukemia (ALL), the most common form of childhood cancer. Therefore, a stringent regulation of Ikaros is of paramount importance for normal lymphocyte ontogeny. Here we provide genetic and biochemical evidence for a previously unknown function of Bruton's tyrosine kinase (BTK) as a partner and posttranslational regulator of Ikaros, a zinc finger-containing DNA-binding protein that plays a pivotal role in immune homeostasis. We demonstrate that BTK phosphorylates Ikaros at unique phosphorylation sites S214 and S215 in the close vicinity of its zinc finger 4 (ZF4) within the DNA binding domain, thereby augmenting its nuclear localization and sequence-specific DNA binding activity. Our results further demonstrate that BTK-induced activating phosphorylation is critical for the optimal transcription factor function of Ikaros.

## Introduction

Bruton's tyrosine kinase (BTK) is a physiologically important kinase that serves as a key regulator of multiple biochemical signal transduction events and biologic responses in B-lineage lymphoid cells throughout B-cell ontogeny [1]–[4]. In B-lineage lymphoid cells, BTK is an essential component of the B-cell signalosome and participates in regulation of cell survival, activation, proliferation, maturation and differentiation [1]–[4]. Additionally, a functional interaction was discovered between BTK and the transcription factor signal transducers and activators of transcription 5A (STAT5A), a molecular regulator of proliferation, differentiation and apoptosis in hematopoietic cells that contributes to interleukin 7-induced B-cell precursor expansion [5]. Recently, it has been discovered that BTK is also expressed in non-lymphohematopoietic cells [6]–[8]. Notably, BTK has been identified as a dual-specificity kinase that not only phosphorylates tyrosine but also serine residues [9]. Specifically, BTK was shown to directly phosphorylate cAMP-responsive Element-binding protein (CREB) at S133 residue [9].

Ikaros (IK) is a zinc finger (ZF)-containing sequence-specific DNA-binding protein that plays an important role in immune homeostasis through transcriptional regulation of the earliest stages of lymphocyte ontogeny by both (a) gene transcriptional activation via efficient transcription initiation and elongation as well as (b) gene repression [10]–[12]. In a recent study, we identified the spleen tyrosine kinase (SYK) as a posttranslational regulator of IK and determined that SYK-induced activating phosphorylation of IK at unique C-terminal serine phosphorylation sites S358 and S361 is essential for its nuclear localization and optimal transcription factor function. [13]. SYK has been shown to cooperate with BTK via the B cell-specific adapter molecule BLNK/SLP-65 in phosphorylation and activation of multiple intracellular effector molecules in the context of B-cell antigen receptor signaling [14]. Furthermore, in the presence of BLNK, SYK directly activates BTK by phosphorylating the Y551 residue in the activation loop of the BTK catalytic domain [14]. Because of the pleiotropic regulatory role of BTK in human B-cell ontogeny, its documented cooperation with SYK in B-cell receptor (BCR)-mediated signaling events, and its role as a downstream effector of SYK, we sought to determine if BTK has any role in post-translational regulation of IK. We now report direct evidence that BTK phosphorylates IK on two unique serine phosphorylation sites S214 and S215 in the close vicinity of its zinc finger 4 (ZF4) within the DNA binding domain, thereby augmenting its nuclear localization and sequence-specific DNA binding activity. Our results further demonstrate that BTK-induced activating phosphorylation is critical for the optimal transcription factor function of IK.

## Materials and Methods

### Cells

Surplus leukemia cells from two patients with newly diagnosed B-lineage ALL were used in subcellular localization studies using confocal imaging. One of the patients was a previously reported infant (<1 year of age) pro-B ALL case (<1 year of age) with very low BTK expression levels and deletion of BTK Exon 16 that results in a frameshift mutation and a truncated catalytic domain [15]. The other patient was a pediatric (<21 years of age) B-lineage ALL patient in relapse with abundant BTK expression and no evidence of BTK deletions [15]. The IRB (CCI) at Children's Hospital Los Angeles (CHLA) (Human Subject Assurance Number: FWA0001914) determined that the use of leukemic cells in our project entitled: “Leukemia Biology Research” did not meet the definition of human subject research per 45 CFR 46.102 (d and f) since it does not include identifiable private information. The CHLA CCI approved the project. The IRB approved project number is CCI-09-00304 (CCI Review Date 12/21/2009, Approval Date: 12/29/09). We also used the human cell lines ALL-N1 (B-precursor ALL xenograft cell line), RAJI (Burkitt's leukemia/lymphoma; ATCC®, CCL-86) and DAUDI (Burkitt's leukemia/lymphoma; ATCC®, CCL-213). In addition, the wildtype DT40 chicken B lymphoma cell line, its BTK-deficient subclone established by homologous recombination knockout of the *btk* gene, and BTK-deficient DT40 cells reconstituted with wildtype *btk* were used as components of a well-established genetic model for B-lineage lymphoid cells [16].

### Reagents

The rabbit polyclonal antibody for IK1 (H-100, sc-13039) was purchased from Santa-Cruz Biotechnology, Inc. (Santa Cruz, CA) for Western blot analysis and fluorescence staining of IK using previously reported procedures [13]. The mouse monoclonal anti-IK and anti-BTK antibodies were prepared in our laboratory and used as previously reported [5], [13], [16]. Goat anti-mouse IgG: Horseradish Peroxidase (HRPO) (M15345) and goat anti-rabbit IgG: HRPO (R14745) antibodies were purchased from Transduction Labs. Green-fluorescent Alexa Fluor 488 dye-labeled secondary antibody Alexa Fluor 488 goat anti-mouse IgG (A-11001) and red-fluorescent Alexa Fluor 568 dye-labeled secondary antibody Alexa Fluor 568 F(ab')_2_ fragment of goat anti-rabbit IgG (A-21069) for confocal microscopy were purchased from Invitrogen (Carlsbad, CA). UltraCruz™ Mounting Medium containing 1.5 μg/ml of 4′, 6-diamidino-2-phenylindole (DAPI) was purchased from Santa-Cruz Biotechnology, Inc. (sc-24941). The mouse monoclonal anti-HA antibody (HA-probe F-7, sc-7392) recognizing an internal region of the influenza hemagglutinin (HA) protein was purchased from Santa-Cruz Biotechnology, Inc. TOTO-3 iodide was obtained from Molecular Probes (Eugene, OR). Molecular weight markers were purchased from Amersham Pharmacia Biotech. All chemicals used were reagent grade or higher. Protein A-Sepharose was purchased from Repligen Corp., Cambridge, MA). Restriction enzymes, and proteinase inhibitors were purchased from Roche (Indianapolis, IN). The siRNA pool used for knock-down of the *btk* gene (Gene ID: 695) (Catalog No. L-003107) included 5′-UGAGCAAUAUUCUAGAUGU-3′, 5′-CAACUCUGCAGGACUCAUA-3′, 5′-GGUGAUACGUCAUUAUGUU-3′, and 5′-GCGGAAGGGUGAUGAAUAU-3′. Controls included the ON-TARGET*plus* Non-Targeting siRNA scrambled control pool (Catalog No. D-001810) and siRNA pool for *ku80* (Gene ID: 7520) (Catalog No. L-010491) including 5′-AAACUUCCGUGUUCUAGUG-3′, 5′-GAGCAGCGCUUUAACAACU-3′, 5′-CGAGUAACCAGCUCAUAAA-3′, and 5′-GCAUGGAUGUGAUUCAACA-3′. All siRNA were purchased from Thermo Scientific Dharmacon, Lafayette, CO, USA. Trypsin was purchased from Promega (USA). All organic solvents except for acetonitrile from VWR (USA) and chemicals at highest available purity were purchased from Sigma Aldrich (St. Louis, MO, USA). MALDI matrix alpha-cyano-4-hydroxycinnamic acid (CHCA) was purchased from Agilent Technologies (Cat. No. G2037A).

### Site-Directed Mutagenesis

The full-length mouse *Ikaros/IKZF1* cDNA (NM_001025597) was subcloned into the pCMV6-Entry precision shuttle vector (Cat# PS100001 Origene, CA, USA) at the restriction sites SgfI and MluI to generate the pCMV6-mIK mammalian cell expression vector. The pCMV6-mIK construct was then used as a backbone vector to generate the mIKS214A_S215A, mIKS168A, and mIKS168A_214A_S215A mutant vectors encoding FLAG-tagged IK proteins with alanine mutations at the BTK phosphorylation sites S168, S214 and S215 using the QuikChange II Site-Directed Mutagenesis Kit from Agilent Technologies (Santa Clara, CA, USA) as previously reported for SYK phosphorylation site mutants [13]. The synthetic oligonucleotide primers that were used to mutate the BTK phosphorylation sites of IK were: S168A-sense, 5′-acatcaagctgcacgcgggtgagaagccc-3′; S168A-antisense, 5′-gggcttctcacccgcgtgcagcttgatgt-3′; S214A-S215A-sense, 5′-gccggagctataaacagcgagccgctttagaggagcataaagag-3′; S214A-S215A antisense, 5′-ctctttatgctcctctaaagcggctcgctgtttatagctccggc-3′. The final vectors were subjected to DNA sequencing with a T7 forward primer 5′-TAATACGACTCACTAT-3′ (Invitrogen, CA) at the DNA Sequencing Core Facility of the University of Southern California. Hek293T cells were transfected with the IK expression vectors using the BioT^Tm^ transfection reagent (Bioland Scientifics, CA). Functional studies were performed 72 hours after transfection. We also used plasmids for the 12A mutant mouse IK protein containing alanine mutations at the 11 CK2 target sites along with the PP1 recognition motif (A465/467) (viz: pcDNA3.1-HA-mIK-13A, 21-23A, 63A, 101A, 294A, 393-394A, 396A, 398A, 465-467A) and the 6D mutant mouse IK protein with combined aspartate phosphomimetic mutations of six N-terminal CK2 phosphorylation sites in our transfections [13], [17]. Hek293T cells were transfected with the IK expression vectors using the BioT^Tm^ transfection reagent (Bioland Scientifics, CA). Functional studies were performed 72 hours after transfection. Site-directed mutagenesis was also performed for 4 specific tyrosine (Y) residues of IK (Y292, Y409, Y493 and Y499) simultaneously to generate the corresponding phenylalanine (F) quarto-mutant that was used as a control in functional assays [13].

### Recombinant Ikaros 1 (IK1) Protein

The cDNAs encoding murine IK1 was cloned into the *E-coli* expression vector pMAL-C2 with the isopropyl-1-thio-beta-galactopyranoside-inducible Ptac promoter to create an in frame fusion between IK1 coding sequence and the 3′end of the E-coli maIE gene, which codes for maltose-binding protein (MBP). *E-coli* strain DH5a was transformed with the generated recombinant plasmid and single transformants were expanded in 5 ml of LB medium (1% tryptone, 1% NaCl, 0.5% yeast extract) containing ampicillin (1000 µg/ml) by overnight culture at 37°C. Expression of the MBP-tagged IK1 was induced with 10 mM isopropyl-1-thio-beta-galactopyranoside. The cells were harvested by centrifugation at 4500×g in a Sorvall RC5B centrifuge for 10 min at 4°C, lysed in sucrose-lysozyme buffer (20 mM Tris, pH 8.0, 150 mM NaCl, 10% sucrose, 1 mM EDTA, 20 mM lysozyme), and further disrupted by sonication. After removal of the cell pellets by centrifugation at 35,000×g for 1 h at 4°C, MBP-tagged IK1 protein was purified from the respective culture supernatant by amylose affinity chromatography, as previously described [13].

### Standard Biochemical Assays

Immunoprecipitations (IP), kinase assays (KA), phospho-amino acid analyses (PAA), and Western blot analysis (WB) using the enhanced chemiluminescence (ECL) detection system (Amersham Pharmacia Biotech) were performed, as described in detail in previous publications [5], [13], [18]. In PAA, the positions of ninhydrin-stained phosphoamino acid standards (phosphoserine [S], phosphothreonine [T], and phosphotyrosine [Y]) are indicated with circles. We used BCL1 cells in stimulation experiments with CD19xCD19 monoclonal antibody homoconjugate (1 µg/ml×15–30 min) to examine the effects of the BTK inhibitor LFM-A13 (alpha-cyano-beta-hydroxy-beta-methyl-N-(2,5-ibromophenyl) propenamide) on BTK-dependent IK serine phosphorylation using standard biochemical assays [5], [13], [18]. LFM-A13 is a rationally designed selective BTK inhibitor that was prepared in our laboratory [19] and it was used at increasing concentrations ranging from 0.5–100 µM to study the effects of BTK inhibition on the DNA binding activity of native IK in BCL1 cells.

### Electrophoretic Mobility Shift Assays (EMSAs)

EMSAs were performed on purified recombinant proteins as well as nuclear extracts from the EBV-transformed human B-lymphoblastoid cell line BCL1 and Hek293T cells, as previously described [13]. Preparation of nuclear extracts was carried out using CHEMICON's Nuclear Extraction Kit (Catalog No. 2900) (Millipore, Billerica, MA, USA) with some modifications. 5 million cells were spun down and washed with 1× phosphate buffered saline (PBS). Cells were resuspended in cytoplasmic buffer containing 0.5 mM DTT and a 1/1000 dilution of Protease Inhibitor Cocktail by gentle inversion and were then incubated on ice for 15 minutes. The cell suspension was centrifuged at 250×g for 5 minutes at 4°C. The cell pellet was resuspended in ice-cold cytoplasmic lysis buffer and disrupted using a 27-gauge needle. The disrupted cell suspension was centrifuged at 8,000×g for 20 minutes at 4°C. The supernatant containing the cytosolic portion of the cell lysate was removed. The remaining pellet containing the nuclear portion of the cell lysate was resuspended in ice-cold nuclear extraction buffer containing 0.5 mM DTT and 1/1000 dilution of Protease Inhibitor Cocktail. The nuclear suspension was gently agitated using an orbital shaker at 4°C for 1 hour and then centrifuged at 16,000×g for 5 minutes at 4°C to isolate the extracted nuclear protein fraction. The protein concentration of extracts was determined by using the Quick Start™ Bradford Protein Assay Kit (Catalog No. 500-0202) (Bio-Rad, Hercules, CA, USA). Oligonucleotide probes for EMSA were purchased from Integrated DNA Technologies (IDT, San Diego, CA, USA) and included *IK-BS1* (5′-TCAGCTTTTGGGAATACCCTGTCA-3′) and *IK*-*BS5* (5′-TCAGCTTTTGAGAATACCCTGTCA-3′) [13]. The *IK-BS1* oligonucleotide probe containing a high-affinity IK1 binding site was end-labeled with [gamma-^32^P]ATP (3000 Ci/mmol) using T4 polynucleotide kinase and purified using a Nuctrap probe purification column (Stratagene). ∼3-μg samples of the nuclear extracts and 1 ng of labeled *IK-BS1* probe (1×10^5^ cpm/ng) were used in the DNA binding reaction. For competition reactions, a 60-fold excess of unlabeled *IK-BS1* probe was added prior to the addition of the labeled IK-BS1 probe. The IK-BS5 oligonucleotide has a single base pair (G>A) substitution at position 3 within the core consensus and does not bind IK [13]. In addition, we used the gamma-satellite probe A (5′-TATGGC GAGGAAAAC TGAAAAAGGTGGAAAATTTAGAAATGT-3′ and 5′-ACATTTCTAAATTTTCCACCTTTTTCAGTTTTCCTCGCCATA-3′) derived from the centromeric gamma-satellite repeat sequences [13]. The gamma-satellite A DNA probe contains two consensus IK binding sites in close proximity to each other and is a target for high affinity binding of wildtype IK and the binding affinity to this probe shows an excellent correlation with the homing of IK to PC-HC. EMSAs were also performed using the Thermo Scientific LightShift Chemiluminescent EMSA Kit (Catalog No. 20148) (Pierce, Rockford, IL, USA) following the manufacturer's protocol [13]. In these experiments, single stranded *IK-BS1* and *IK-BS5* oligonucleotides were biotin-labeled using the Biotin 3′ End Labeling Kit (Catalog No. 89818) (Pierce, Rockford, IL, USA). 100 nmols of unlabeled oligonucleotides were incubated for 30 minutes at 37°C in a reaction mixture containing 1X TdT Reaction Buffer (500 mM cacodylic acid, 10mM CoCl_2_, 1mM DTT, pH 7.2), 0.5 μM Biotin-11-UTP and 0.2 Units of TdT. 0.2 M EDTA was added to stop the reaction and biotin-labeled oligonucleotides were extracted using chloroform: isoamyl alcohol (24∶1). Single-stranded biotinylated oligonucleotides were duplexed by mixing together equal amounts and incubating for 1 hour at room temperature. Each binding reaction for EMSA included 10× Binding Buffer (100 mM Tris, 500 mM KCl, 10 mM DTT), 2.5% Glycerol, 5 mM MgCl_2_, 50 ng Poly (dI*dC), 0.05% NP-40, ∼0.4 (1×) or ∼4 µg (10×) nuclear protein extract (NE), and 20 fmols of the biotin-labeled duplexed probe in a total volume of 20 µl. Binding reactions were performed at room temperature for 20 minutes. A 6% non-denaturing polyacrylamide gel was pre-run during the 20 min incubation time at 200 V in pre-chilled 0.5× TBE buffer (89 mM Tris base, 89 mM boric ×cid, 1 mM EDTA, pH ∼8.0). 5× loading buffer was added to each reaction sample and samples were loaded onto a polyacrylamide gel. Samples were electrophoresed at 100 V and transferred at 380 mA (∼50 V) for 30 minutes to a Biodyne B Nylon Membrane (Catalog No. 77016) (Thermo Scientific, Rockford, IL, USA) soaked in 0.5× TBE buffer. When the transfer was completed, biotin-labeled DNA was cross-linked to the membrane at 120 mJ/cm^2^ using a Spectrolinker XL-1000 UV cross-linker with 254-nm UV light bulbs. The biotin-labeled DNA was detected using a stabilized streptavidin-horseradish peroxidase (HRP) conjugate and a highly sensitive chemiluminescent substrate according to the manufacturer's instructions [13]. The membrane was exposed to X-ray film and developed with a film processor.

### RT-PCR Analysis of Ikaros Target Genes

Reverse transcription (RT) and polymerase chain reaction (PCR) were used to evaluate the expression levels of previously published and validated IK target genes [13]. Total cellular RNA was extracted from cells using the QIAamp RNA Blood Mini Kit (Catalog No. 52304) (Qiagen, Santa Clarita, CA, USA) following the manufacturer's instructions. Oligonucleotide primers were ordered from Integrated DNA Technologies (IDT, San Diego, CA, USA) to amplify a 244 bp region of the *ITGA4* transcript (Gene ID 3676, Forward/Reverse: 5′- GAGTGCAATGCAGACCTTGA -3′/5′- TGGATTTGGCTCTGGAAAAC -3′), a 236-bp region of the *KIF23* transcript (Gene ID 9493, Forward/Reverse: 5′- CGGAAACCTACCGTGAAAAA -3′/5′- AGTTCCTTCTGGGTGGTGTG -3′), a 168 bp region of the *TNFAIP8L2* transcript (Gene ID 79626, Forward/Reverse: 5′- GGCACTTAGCTTTGGTGAGG -3′/5′- AGCAGACCTGGGTCAGAGAA -3′), and a 209-bp region of the *EIF4E3* transcript (Gene ID 317649, Forward/Reverse: 5′- CCGCAGCAGATGATGAAGTA-3′/5′- GTGTTTTCCACGTCCACCTT-3′) [13]. The QIAGEN One-Step RT-PCR Kit (Catalog No. 210212) (Qiagen, Santa Clarita, CA, USA) was used following manufacturer's instructions to amplify the target PCR products. The conditions were 1 cycle ×(30 min 50°C, 15 min 95°C) and 35 cycles ×(45 sec 94°, 1 min 60°, 1 min 72°). PCR products were separated on a 1.2% agarose gel and visualized after ethidium bromide staining using a UVP Epi Chemi II Darkroom Transilluminator.

### Genes involved in lymphoid-lineage affiliated transcriptional program controlled by Ikaros

Twenty lineage-affiliated genes *(FLT3, NOTCH1, LTB, BTLA, CD52, CLNK, IL7R, CCR9, DNTT, IGJ, SATB1, SOX4, RUNX2, MEF2C, RAG1, HMGA2, CNN3, PTGER2, ETS1, CSF1R)* implicated in lymphoid priming were compiled from mouse studies for evaluation of their expression levels in lymphocyte precursors from primary leukemia specimens of children with ALL [13].

### Confocal Laser Scanning Microscopy

Subcellular IK localization and co-localization studies using immunofluorescence and spinning disk confocal microscopy were performed as previously described [13]. In experiments using primary lymphocyte precursors from BTK-abundant vs. BTK-deficient ALL patients, confocal microscopy was performed using a BioRad MRC-1024 Laser Scanning Confocal Microscope (BioRad, Hercules, CA, USA) equipped with a krypton/argon mixed gas laser and mounted on a Nikon Eclipse E800 series upright microscope equipped with high numerical objectives. TOTO-3 iodide from Molecular Probes (Eugene, OR) was used for nuclear staining. Confocal images were obtained using a Nikon 60×(NA 1.4) objective and Kalman collection filter. During confocal imaging in other experiments, slides were imaged using the PerkinElmer Spinning Disc Confocal Microscope and the PerkinElmer UltraView ERS software (Shelton, CT) or the Volocity V5.4 imaging software (PerkinElmer, Shelton, CT). The coverslips were fixed with ice-cold MeOH at −20 C for 10 minutes. The fixed cells were permeabilized and their non-specific antibody binding sites blocked with 0.1% Triton X-100 and 10% goat serum in PBS for 30 minutes, respectively. In order to detect and localize the BTK and IK proteins, cells were stained with an in house mouse monoclonal anti-BTK antibody, an in house mouse monoclonal anti-IK antibody or a mouse monoclonal anti-HA antibody (HA-probe F-7, sc-7392) recognizing an internal region of the influenza hemagglutinin (HA) protein as primary antibodies for 1 hour at room temperature. Cells were washed with PBS and incubated with appropriate secondary antibodies conjugated with either Alexa Fluor 488 (Cat #: A11001, Invitrogen, Carlsbad, CA) or Alexa Fluor 568 (Cat #: A21069, Invitrogen) for 1 hour. To detect the FLAG-tagged wildtype and BTK-phosphorylation site mutant IK proteins, cells were incubated with a monoclonal mouse anti-DDK (TA50011–100, Origene) and a secondary antibody conjugated with Alexa Fluor 488. Cells were then washed with PBS and counterstained with the DNA-specific nuclear dye DAPI. The coverslips were inverted, mounted onto slides in Vectashield (Vector Labs, Burlinghame, CA) to prevent photobleaching, and sealed with nail varnish. UltraCruz Mounting Medium containing 1.5 μg/ml of 4′,6-diamidino-2-phenylindole (DAPI) was purchased from Santa Cruz Biotechnology, Inc. (Santa Cruz, CA). The coverslips were inverted, mounted onto slides in Vectashield (Vector Labs, Burlinghame, CA) to prevent photobleaching, and sealed with nail varnish. Fluorescent cells were imaged using the PerkinElmer Ultraviewer Confocal Dual Spinning Disc Scanner (Shelton, CT). Settings Photos were analyzed and processed using the Velocity ver5.4 imaging visualization software.

### Recombinant Baculovirus Expression System

Recombinant murine BTK and recombinant BTK kinase domain were produced in a baculovirus expression system and purified as previously described in detail [19], [20].

### Molecular Model of BTK-Phosphorylated Ikaros Segment

We constructed a molecular model of BTK and the BTK-phosphorylated segment of IK1 (IK1 peptide, residues 203–224) based on the X-ray crystal structure of BTK (PDB entry: 3PIZ) and by using comparative homology modeling [13], [21]–[24]. The structure of IK peptide was built by Modeller (21) and homologous crystal templates (PDB entry: 1MEY and 2I13). Conformation and the primary position of ATP were selected by homology modeling through structural superimposition between the crystal structures of insulin receptor tyrosine kinase (PDB entry: 1IR3) and BTK using Sybyl6.8 (Tripos, St. Louis, MO). Then, the target IK peptide was “grafted” into the catalytic site of BTK by using the computational protein-protein docking web server GRAMM (22). After obtaining the initial structure of the BTK-ATP-IK peptide complex, the position of the peptide within a 6.5 Å radius around the catalytic site [23] was optimized by energy minimization using the AMBER force field implemented in the Sybyl with the following parameters: (i) a distance-dependent dielectric function, (ii) non-bonded cutoff 8Å, (iii) Gasterger-Hückel charges for ATP, (iv) Amber charges for the protein and the peptide. The structure was minimized first by applying the simplex method, followed by the use of the Powell method to the energy gradient <0.05 kcal/(mol⋅Å).

### Matrix-Assisted Laser Desorption/Ionization–Time-Of-Flight (MALDI-TOF) Mass Spectrometry

The services of Applied Biomics (Hayward, CA, USA) were used for the identification of phosphorylation sites by MALDI-TOF/TOF following a standard protocol. In brief, after the *in vitro* kinase reaction with recombinant murine BTK, phosphorylated recombinant murine IK1 samples were digested in solution overnight at 37°C with trypsin. Supel-Tips (Sigma-Aldrich) were used for phosphopeptide enrichment. Tryptic peptides were desalted and concentrated using the Millipore C18 reverse phase Zip-Tips column (ZTC 18S096, Millipore, USA), eluted in 0.5 μL of matrix solution (α-cyano-4-hydroxycinnamic acid [5 mg/mL in 50% acetonitrile, 0.1% trifluoroacetic acid, 25 mmol/L ammonium bicarbonate]), and spotted on the MALDI plate (model ABI 01-192–6-AB). MALDI-TOF MS (matrix-assisted laser desorption/ionization–time-of-flight MS) was performed on an AB Sciex Proteomics Analyzer (AB Sciex, Foster City, CA, USA). MS spectra were acquired in reflection positive ion mode, averaging 4000 laser shots/ per spectrum. A virtual digest was done by submitting protein sequences of interest to University of California–San Francisco Protein Prospector (http://prospector.ucsf.edu/ prospector/mshome.htm). The MS precursors matching the virtual digest were submitted for collision induced dissociation (CID) fragmentation. Peptide masses and associated CID spectra were submitted to GPS Explorer workstation equipped with MASCOT search engine (Matrix Science) to search the database of Swiss-Prot. Candidates with either a protein score confidence interval percentage or ion confidence interval percentage of >95% were considered significant. The spectra of all peptides containing potential phosphorylation sites were manually evaluated for the loss of phosphate.

### Bioinformatics and Statistical Analysis of Gene Expression Profiles

In the analyses of gene expression profiles of lymphocyte precursors with high vs. low BTK/IK expression levels, we focused our analysis on validated IK target genes [13]. The publically available archived GSE32311 database was used to compare gene expression changes in CD4^+^CD8^+^ double-positive wild type (N = 3; GSM800500, GSM800501, GSM800502) vs. *Ikaros/IKZF1* null mouse thymocytes (N = 3, GSM800503, GSM800504, GSM800505) from the same genetic background of (C57BL/6 x129S4/SvJae) [13]. Gene expression changes were screened utilizing probe level RMA signal intensity values from the mouse 430_2.0 Genome Array. In order to identify the gene signatures for up-regulated and down-regulated transcripts in *Ikaros/IKZF1* knockout mice, we filtered changes greater than 2 fold and T-test P-values less than 0.05 (T-test, Unequal Variances, Excel formula). Application of this filter identified 1158 transcripts representing 924 genes that were down-regulated in *Ikaros/IKZF1* null mice with a subset of 201 transcripts representing 137 genes exhibiting >2-fold decreased expression levels. By cross-referencing this IK-regulated gene set with the archived CHiPseq data (GSM803110) using the Integrative Genomics Browser [25], we identified 45 IK target genes that harbored IK binding sites (13). The Gene Pattern web based software was utilized (http://www.broadinstitute.org/cancer/software/genepattern) to extract expression values for human lymphocyte precursors from the National Center for Biotechnology Information (NCBI) Gene Expression Omnibus (GEO) database. We compiled 1104 primary leukemia specimens from pediatric ALL patients (GSE3912, N = 113; GSE18497, N = 82; GSE4698, N = 60; GSE7440, N = 99; GSE13159, N = 750). We focused our analysis on 45 validated mouse IK target genes as well as 20 lymphoid-priming genes [13] on a subset of 884 samples obtained from B-lineage ALL patients.

Human orthologs of the mouse genes were identified by interrogating the gene symbols of the mouse genes using the curated online repository of HGNC-approved gene nomenclature (http://www.genenames.org/). For each study, the gene expression values were transformed into standard deviation units calculated from the mean and standard deviation expression values for all the samples in each study. Standardized values compiled from the 5 studies were rank ordered according to the expression of a *BTK* transcript (205504_at). Prospective power analysis was utilized to determine the Standard Deviation cut-off for “high *BTK/Ikaros* expression” and “low *BTK/Ikaros* expression” samples in the data sets. We set the unadjusted critical P-value at 2.5×10^−6^ to control for False Positive Rate (FPR) at 0.05 in order to detect significant differences in any one of the *BTK/Ikaros* transcripts out of approximately 20,000 transcripts common across the 5 Affymetrix platforms. At this critical P-value, a total sample size greater than 254 would be sufficient to detect a difference of 1 standard deviation unit with 99.9% power for *BTK/Ikaros* transcripts. Therefore, samples were assigned to the “high *BTK* expression” group if their expression level was >0.5 standard deviation unit higher than the mean expression level (N = 369) and to the “low *BTK* expression” group if their expression level was >0.5 standard deviation unit lower than the mean expression level (N = 125). Similar calculation utilizing the averaged standardized expression values for *IKZF1* probesets (205038_at, 205039_s_at, 216901_s_at, 227344_at and 227346_at) were rank ordered for 1104 ALL samples, and samples with expression values greater than 0.5 standard deviation units were assigned “high *IKZF1* expression” (N = 258) and those with less than −0.5 standard deviation units were assigned “low *IKZF1* expression” (N = 247). T-tests were performed using standardized expression values combined from 5 datasets (2-sample, Unequal variance correction, p-values<0.05 deemed significant) revealing an intersect of 34 transcripts representing 24 IK target genes and 15 transcripts representing 12 genes for lymphoid-priming genes that were significantly up-regulated in both high *BTK* and high *Ikaros/IKZF1* expression groups. We used a one-way agglomerative hierarchical clustering technique to organize expression patterns using the average distance linkage method such that genes (rows) having similar expression across patients were grouped together (average distance metric). Dendrograms were drawn to illustrate similar gene-expression profiles from joining pairs of closely related gene expression profiles, whereby genes joined by short branch lengths showed the most similarity in expression profile across all samples [13]. Standardized expression values (SD units) for *BTK* probeset (205504_at) compiled from the 5 studies comprising of 1104 primary leukemia specimens from pediatric acute lymphoblastic leukemia (ALL) patients (GSE3912, N = 113; GSE18497, N = 82; GSE4698, N = 60; GSE7440, N = 99; GSE13159, N = 750) were tested for correlation with the expression of 3 *Ikaros*/*IKZF1* probesets common in all 5 studies (205038_at, 205039_s_at, 216901_s_at). By cross-referencing IK-regulated gene set (GSE323211) with the archived CHiPseq data (GSM803110) the location of potential IK binding sites for validated IK target genes *Itga4* (NM_010576; chr2:79095583–79173271), *Eif4e3* (NM_025829; chr6:99575131–99616765), *Kif23* (NM_024245; chr9:61765085–61794606) and *Tnfaip8l2* (NM_027206; chr3:94943443–94946282) was visualized in the mouse mm9 reference database using the Integrative Genomics Browser.

### siRNA Transfections

Hek293T cells (ATCC # CRL-111268) were cultured in 6-well plate with DMEM medium supplemented with 10% FBS. They were transfected with siRNA after reaching 70–80% confluence using ON-TARGET*plus* SMARTpool siRNA and DharmaFECT Transfection Reagent 4 (Catalog No. T-2004) (Thermo Scientific Dharmacon, Lafayette, CO, USA). Transfections were performed following the manufacturer's instructions [13]. 50 nM of ON-TARGET*plus* SMARTpool siRNA and transfection reagent were mixed in antibiotic-free complete media and added to adherent cells. In experiments aimed at evaluating the effects of *btk* knockdown on native IK function, cells were transfected with the human BTK specific siRNA (On-Target*Plus* ^Tm^ Cat# L-003107). Control siRNA included ON-TARGET*plus* Non-targeting siRNA scrambled control pool as well as Ku80 siRNA (in experiments aimed at documenting BTK siRNA-induced selective BTK protein depletion) as negative controls. 72 hours after transfection with the siRNA, Hek293T cells were examined for the subcellular localization, DNA binding activity, and transcription factor function of native IK. In some experiments, siRNA-transfected Hek293T cells (3×10^5^ cells/sample) were seeded onto poly-L-lysine coated glass coverslips (22×22 mm) after 48 hours, allowed to adhere to the coverslips placed in 6-well plates by incubation overnight and then transfected with expression vectors for the 12A or 6D mutant mouse IK proteins (2 µg/well) using the BioT^Tm^ transfection reagent (Bioland Scientifics, CA). At 48 hours after the second transfection, Hek293T cells were examined for the subcellular localization of the recombinant mutant IK proteins.

## Results

### Correlation between the expression levels of *BTK* and lymphoid priming genes

IK has been shown to direct lymphoid priming during the earliest stages of lymphocyte ontogeny by upregulating the expression of specific genes [10]–[12]. In our search for potential partners of IK, we discovered that 15 transcripts representing 12 IK-regulated lymphoid priming genes were significantly upregulated in human lymphocyte precursor cells from primary bone marrow specimens of pediatric patients with B-lineage ALL expressing high levels of both *IKZF1/IK* and *BTK* genes (**Figure S1 in File S1**). The 5 most significantly up regulated genes in specimens with high BTK expression were *MEF2C* (3 transcripts ranging from 0.71 to 1.04 SD units, P = 4.3×10^−19^ to 9.7×10^−43^), *DNTT* (0.93 SD Units, P = 5.6×10^−24^), *RAG1* (0.72 SD units, P = 2.5×10^−18^), *IL7R* (0.65, P = 1.9×10^−14^) and *FLT3* (0.62, P = 1.6×10^−13^). Hierarchical cluster analysis revealed a subset of 2 genes (*MEF2C (3 transcripts)* and *FLT3)* that were highly co-regulated with BTK (**Figure S1A in File S1**). Furthermore, highly significant correlations were observed for *IKZF1* probesets plotted against the *BTK* probeset: 205038_at (Correlation Coefficent  = 0.27, T-value  = 9.24, P<0.0001), 205039_s_at (Correlation Coefficent  = 0.19, T-value  = 6.47, P<0.0001) and 216901_s_at (Correlation Coefficent  = 0.19, T-value  = 6.37, P<0.0001) (**Fig. S1B in File S1**). The apparent impact of differential *BTK* expression levels (viz.: high *BTK*-low *BTK*, **Figure S1C in File S1)** on the transcript levels of these lymphoid priming genes (highest ranking 11 out of 15 transcripts), as measured by standard deviation units, was more pronounced than the impact of differential IK expression levels (viz.: high *IKZF1*-low *IKZF1*, **Figure S1C in File S1**) (Paired T-test on 11 transcripts, Mean Difference  = 0.21, Df  = 10, T-value  = 5.095, P = 0.0005; Wilcoxon Rank, Median Difference  = 0.24, P = 0.002). While the observed correlation between *BTK* and *IKZF1* transcript levels suggest transcriptional co-regulation by an as yet unknown mechanism, the striking dependency of the expression levels of IK-regulated lymphoid priming genes on the *BTK* expression levels prompted the hypothesis that BTK may also be involved in the regulation of IK function.

### Correlation between the expression levels of BTK and validated Ikaros target genes harboring Ikaros binding sites

We further explored the role of BTK in regulation of the transcription factor function of native IK in human lymphocyte precursors by comparing the expression levels of 45 recently validated IK target genes harboring IK binding sites [13] in primary samples of lymphocyte precursors from B-lineage ALL patients (**Figure S2 in File S1)**. T-tests were performed using standardized expression values revealing an intersect of 34 transcripts representing 24 genes that were significantly upregulated in both high *BTK* and high *IKZF1* expression groups (**Figure S2A in File S1**). As with the lymphoid priming genes, the impact of differential *BTK* expression levels (viz.: high *BTK*-low *BTK*) on the transcript levels of the IK target genes, as measured by standard deviation units, was more pronounced than the impact of differential IK expression levels (viz.: high IKZF1-low IKZF1, **Figure S2B in File S1;** Paired T-test on 34 transcripts, Mean Difference  = 0.08, Df  = 33, T-value  = 2.778, P = 0.0089; Wilcoxon Rank, Median Difference  = 0.19, P  = 0.0089). While the observed correlation between the transcript levels of *BTK* and IK target genes suggested that BTK may be involved in IK regulation, it could not be taken as evidence that BTK is a regulator of IK because (i) several alternative scenarios could account for this association, including transcriptional co-regulation and (ii) BTK function is subject to posttranslational regulation by its negative regulators and miR-185. Therefore, we took a systematic stepwise approach to carefully examine the physical and functional interactions between BTK and IK.

### Subcellular co-localization and physical association of BTK and Ikaros proteins

**Figure 1:**
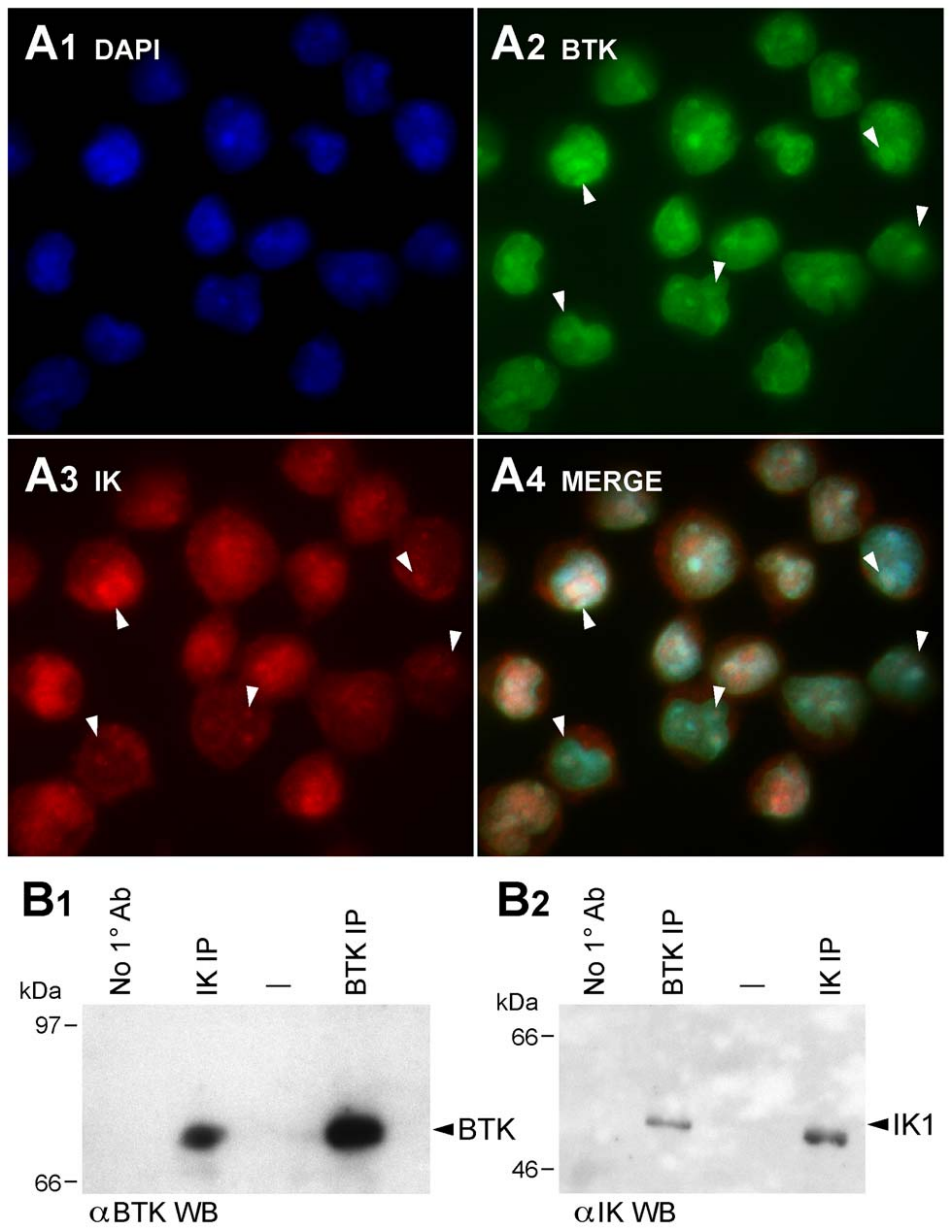
Co-localization and Physical Interactions of Native Ikaros and BTK Proteins in Human Cells. [A] Nuclear co-localization of Native IK and BTK. ALL-N1 cells were fixed and stained with polyclonal rabbit anti-IK1 (primary Ab)/Alexa Fluor 568 F(ab')_2_ fragment of goat anti-rabbit IgG (secondary Ab) (red) and mouse anti-BTK MoAb (primary Ab)/Alexa Fluor 488 goat anti-mouse IgG (secondary Ab) (green) antibodies. Nuclei were stained with blue fluorescent dye 4′,6-diamidino-2-phenylindole (DAPI). MERGE panels depict the merge three-color confocal image showing co-localization of IK1 and BTK in DAPI-stained nucleus as magenta immunofluorescent foci (System magnification: 315×). Representative foci of colocalization are indicated with white arrowheads. [B] Co-immunoprecipitation of Native IK and BTK. B1 depicts the results of the BTK Western blot analysis of the IK and BTK immune complexes immunoprecipitated (IP) from ALL-N1 cells. B.2 depicts the results of the IK Western blot analysis of the BTK and IK immune complexes from the same cells. Controls included immunoprecipitations performed without using a primary (1^0^) antibody.

While BTK is predominantly cytoplasmic and IK is predominantly nuclear, high-resolution fluorescence microscopy demonstrated that native IK and BTK are partially co-localized in the nucleus as well as cytoplasm of human B-lineage lymphoid cell lines ALL-N1 (**Figure 1A**), RAJI and DAUDI (**Figure S3A in File S1**). In co-immunoprecipitation experiments using Triton X-100 whole cell lysates from these cell lines, IK immune complexes contained BTK (**Figure 1B** **1**; **Figure S3B1 in File S1**) in addition to IK (**Figure 1B** **2**, **Figure S3B.2 in File S1**) and likewise BTK immune complexes contained IK (**Figure 1B** **2**, **Figure S3C1 in File S1**) in addition to BTK (**Figure 1B** **1**, **Figure S3C2 in File S1**). These results suggested that native IK might constitutively associate with native BTK.

### BTK regulates DNA binding activity, transcription factor function, and subcellular localization of native Ikaros

**Figure 2:**
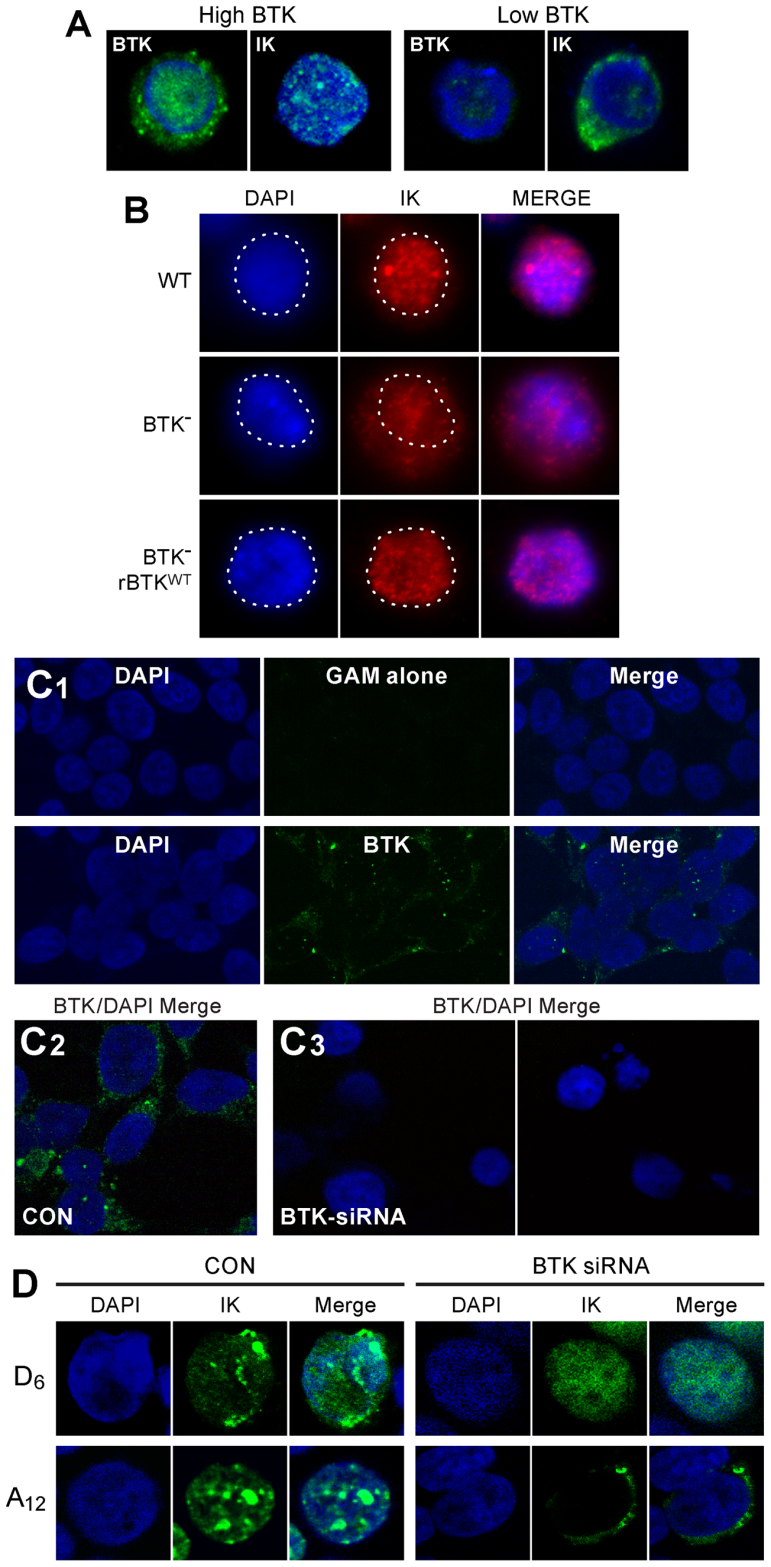
BTK Expression Levels Control Nuclear Localization of Ikaros. [**A**] Cells were stained with mouse monoclonal antibody (mAb) against human BTK and an anti-IK mouse mAb, which was raised against murine IK and which cross-reacts with human IK. TOTO-3 was used for nuclear staining. Depicted are the confocal two-color (green/blue: BTK/TOTO-3 and IK/TOTO-3) merge images of abundantly BTK^+^ (high BTK) leukemic cells from a pediatric B-precursor ALL case vs. leukemic cells with low BTK expression level from an infant B-precursor ALL case. Nuclear staining for IK was observed in the confocal fluorescence images of primary leukemic B-cell precursors with high BTK expression level. By contrast, leukemic cells with low BTK expression levels showed an aberrant, predominantly cytoplasmic localization of IK (System Magnification: 500×). [**B**] The DT-40 cell line and its subclones were stained with the rabbit polyclonal, H-100 (sc-13039) antibody against the N-terminus of IK. Depicted are the confocal two-color (red/blue) IK/DAPI merge images of wildtype (WT) DT40 cells, BTK-deficient DT40 cells, and BTK-deficient DT40 cells reconstituted with wildtype *btk*. The contour of the DAPI-stained nuclei (blue) was marked with a dotted line and shows a significant amount of IK protein (red) outside the nucleus of the BTK^−^ DT40 cells. [**C1**] Upper panel: Depicted are the confocal single-color and two-color merge images of control Hek293T cells stained with the secondary goat anti-mouse (GAM) antibody and DAPI. No false positive green fluorescence was detected (System Magnification: 630×). Lower panel: Depicted are the confocal single-color and two-color (green/blue: BTK/DAPI) merge images of test Hek293T cells stained with anti-BTK/goat-anti-mouse (GAM) antibody combination and DAPI. BTK (green fluorescence) was localized primarily in the cytoplasm of Hek293T cells around the DAPI-stained blue nucleus. [**C2**] Left panel: Merge confocal images of untreated control Hek293T cells two-color stained with anti-BTK/goat-anti-mouse (GAM) antibody combination and DAPI. Right panel: Merge confocal images of BTK-siRNA transfected Hek293T cells two-color stained with anti-BTK/goat-anti-mouse (GAM) antibody combination and DAPI 72 h post-transfection. (System Magnification: 630×). BTK depletion was confirmed by Western blot analysis (see Figure 3) [**D**] Confocal images of Hek293T cells expressing the HA-tagged mutant IK proteins D_6_ or A_12_ stained with the mouse monoclonal anti-HA antibody (HA-probe F-7)(primary Ab)/Alexa Fluor 488 goat anti-mouse IgG (secondary Ab) (green) antibody and blue fluorescent DNA dye 4′, 6-diamidino-2-phenylindole (DAPI) following treatment with BTK siRNA (50 nM) vs. PBS (CON). (System Magnification: 630×).

By using confocal laser scanning microscopy, we next examined the subcellular localization of IK in BTK-deficient human lymphocyte precursors. Confocal fluorescence images of primary leukemic B-cell precursors from a control pediatric B-cell precursor ALL case expressing high levels of BTK exhibited normal punctate nuclear staining consistent with the localization of IK to PC-HC. By contrast, primary leukemic cells from an infant B-cell precursor ALL patient with very low protein levels of BTK and aberrant BTK transcripts characterized by deletion of exon 16 that results in a truncating frameshift mutation starting at amino acid #523 within the catalytic domain with 4 novel amino acids before the stop codon [15] showed an aberrant, predominantly cytoplasmic localization of IK (**Figure 2A**). Since the nuclear localization of IK is determined by its DNA binding activity, these results uniquely indicated that the IK-BTK interaction might favorably affect the DNA binding activity of native IK in primary human lymphocyte precursors. We next sought genetic evidence for a regulatory role of BTK in nuclear localization of native IK in lymphoid cells using BTK-deficient DT40 chicken B-cell lymphoma clones that were established by homologous recombination knockout [16]. When analyzed by high-resolution fluorescence microscopy, BTK-deficient DT40 cells exhibited an abnormal IK localization profile with much of the IK protein found in the cytoplasm in contrast to the normal speckled nuclear staining pattern for IK in wildtype DT40 cells. (**Figure 2B**). The abnormal subcellular localization of IK in the BTK-deficient DT40 cells was the direct result of lack of BTK as evidenced by the fact that BTK^−^ DT40 cells reconstituted with wild-type human BTK showed a normal nuclear localization of IK (**Figure 2B**). These findings further indicated that BTK plays a role in nuclear localization of native IK.

**Figure 3:**
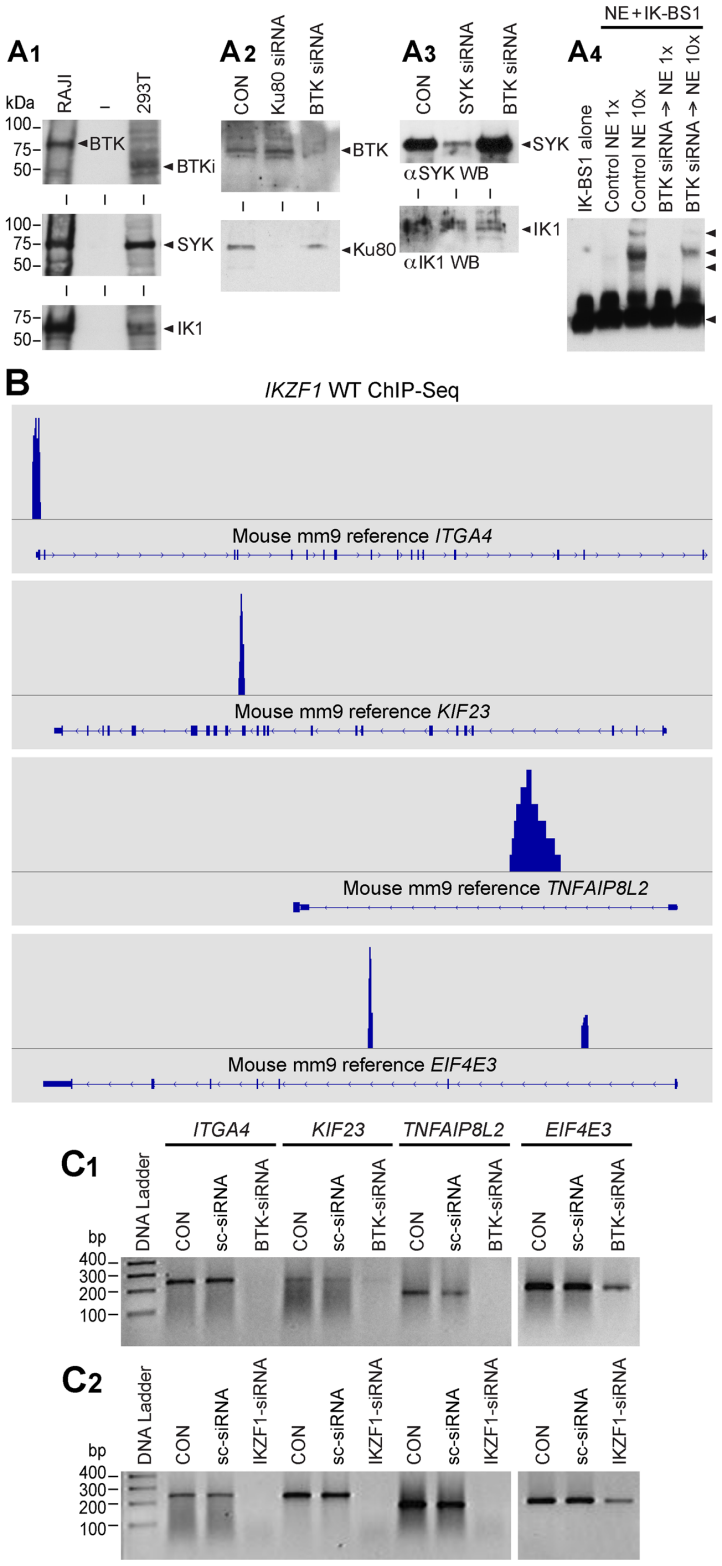
BTK Expression Levels Control DNA Binding Activity and Transcription Factor Function of Ikaros. [**A1**] BTK Western Blot Analysis of RAJI and Hek293T cells. RAJI cells expressed predominantly the 77 kDa isoform of BTK, whereas Hek293T cells expressed predominantly a 65 kDa isoform of BTK (labeled as BTKi). Both RAJI and Hek293T cells express SYK and IK1. [**A2**] Upper panel: BTK Western blot analysis of whole cell lysates from Hek293T cells treated with medium only (CON), BTK siRNA or Ku80 siRNA that was used as a control. Each siRNA was used at a 50 nM concentration. BTK siRNA (but not Ku80 siRNA) resulted in depletion of BTK protein without a decrease in the amount of IK protein. Lower panel **:** Ku80 Western blot analysis of whole cell lysates from Hek293T cells treated with medium only (CON), BTK siRNA or Ku80 siRNA. BTK siRNA did not affect the expression level of the control protein Ku80. In contrast, Ku80 siRNA resulted in depletion of Ku80 protein. [**A3**] Additional Controls. SYK vs. IK Western blot analysis of whole cell lysates from 293T cells treated with medium only (CON), SYK siRNA or BTK siRNA [13]. BTK siRNA did not cause a decrease in the amount of SYK or IK proteins [modified from Figure 2 of our recent open access article published in PNAS [13]. [**A4**] EMSAs were performed on nuclear extracts (NE) from untreated control (CON) Hek293T cells as well as Hek293T cells treated for 72 h with BTK siRNA (50 nM) using biotin-labeled DNA probe IK-BS1. Both 0.4 µg (1×) and 4 µg (10×) amounts of NE were used. IK activity was measured by the electrophoretic mobility shifts of the biotin-labeled IK-BS1 probe, representing IK-containing nuclear complexes (indicated with arrow heads). The position of the probe is also indicated with an arrowhead at the bottom of the gel. The biotin-labeled DNA was detected using a streptavidin-horseradish peroxidase conjugate and a chemiluminescent substrate. The membrane was exposed to X-ray film and developed with a film processor. [**B**] IK binding sites of validated IK target genes. By cross-referencing IK-regulated gene set (GSE323211) with the archived CHiPseq data (GSM803110) the location of potential IK binding sites for validated IK target genes *Itga4* (NM_010576; chr2:79095583–79173271), *Eif4e3* (NM_025829; chr6:99575131–99616765), *Kif23* (NM_024245; chr9:61765085–61794606) and *Tnfaip8l2* (NM_027206; chr3:94943443–94946282) was visualized in the mouse mm9 reference database using the Integrative Genomics Browser. [**C**] RT-PCR was used to examine the expression levels of randomly selected IK target genes after 72 h treatment with medium alone (CON), 50 nM scrambled siRNA (sc-siRNA), *IKZF1* siRNA vs. *BTK* siRNA. C1: Expression levels of 4 randomly selected IK target genes were reduced by siRNA-mediated depletion of BTK, whereas treatment with sc-siRNA had no such effect. C2: Included as a positive control, *IKZF1* siRNA also abrogated or reduced the expression of all 4 IK target genes.

Primary B-cell precursors and B-lineage lymphoid B-cell lines do not permit reproducible transfection with siRNA to achieve significant knock-down at protein level. Hek293T cell line that is easily and reproducibly transfectable was found to be both BTK-positive (**Figure 2C** **1, 2C2**) and IK positive [13] (**Figure 3A** **1**) and therefore provides provides a suitable mammalian cell model for genetic experiments aimed at studying the role of BTK in IK regulation using BTK-specific siRNA. In order to document the significance of BTK to the function of IK, we examined the effects of BTK depletion by RNA interference on IK-specific DNA binding activity in nuclear extracts from human Hek293T cells using EMSAs with the biotin-labeled IK-BS1 oligonucleotide probe containing a high-affinity IK binding site. The selective depletion of BTK by specific BTK siRNA was documented using confocal microscopy (**Figure 2C** **3**) and Western blot analysis (**Figure 3A** **2**). BTK siRNA markedly reduced BTK protein levels (**Figure 3A** **2**) without affecting the expression levels of the IK or SYK proteins [13] (**Figure 3A** **3**). While control Ku80 siRNA caused Ku80 depletion without affecting the BTK protein expression level, BTK siRNA caused BTK depletion without affecting Ku80 protein expression level (**Figure 3A** **2**). We recently reported that SYK siRNA (but not scrambled control siRNA) abolished the DNA binding activity of native IK [13]. Notably, treatment with BTK siRNA also abolished the DNA binding activity of native IK (**Figure 3A** **4**).

IK function, stability, and subcellular localization are affected by casein kinase 2 (CK2) induced phosphorylation [17], [26]. CK2-mediated phosphorylation of IK at 11 previously published serine/threonine phosphorylation sites has been shown to reduce the DNA binding activity of IK [17], [26]. Protein phosphatase PP1 binds and activates IK by dephosphorylating it on CK2 phosphorylated residues [17]. The combined aspartate phosphomimetic mutation of six N-terminal inhibitory CK2 phosphorylation sites (viz.: 13D+21D+23D+63D+101D+294D) causes an abnormal hyperactive nuclear localization of IK when overexpressed in Hek293T cells, as characterized by both speckled and diffuse nuclear IK fluorescence staining (**Figure 2D** **, upper panel**). siRNA-mediated BTK depletion totally abrogated the speckled staining indicative of residual PC-HC localization (**Figure 2D** **, upper panel**). IK mutant 12A containing alanine mutations at the 11 CK2 target sites for inhibitory phosphorylation along with the PP1 recognition motif (A465/467) is able to bind DNA with high affinity [13], [16] and exhibits a normal PC-HC localization when overexpressed in Hek293T cells, as reflected by a speckled nuclear staining pattern (**Figure 2D** **, lower panel**). Notably, siRNA-mediated depletion of native BTK completely blocks the nuclear localization of IK mutant 12A in Hek293T cells, as evidenced by a strictly cytoplasmic staining pattern (**Figure 2D** **, lower panel**). Thus, alanine substitution of CK2-phosphorylated inhibitory serine residues fails to restore the DNA binding activity of IK in the absence of BTK. These results are reminiscent of the effects of SYK-depletion [13] and indicate that BTK-mediated activation of IK is important for its normal PC-HC localization in the nucleus and capable of overriding its CK2-mediated inhibition.

To formally document the role of BTK in the regulation of IK transcription factor function, we examined the effects of BTK depletion by RNA interference on validated IK target gene expression in human Hek293T cells using RT-PCR. Notably, the expression levels of all 4 randomly selected IK target genes (viz.: *ITGA4, MDM1, EIF4E3, KIF23, TNFAIP8L2*) that have direct IK binding sites (**Figure 3B**) were markedly reduced by siRNA-mediated depletion of BTK (**Figure 3C** **1**). Similar results were obtained with siRNA-mediated depletion of IK as well [13] (**Figure 3C** **2**). In contrast, treatment with scrambled siRNA (included as a negative control) had no such effect (**Figure 3C** **1, C2**). The striking BTK-dependency of the IK target gene expression levels demonstrates that BTK plays a critical role in regulation of the native IK function.

### Inhibition of BTK prevents IK serine phosphorylation and reduces IK DNA binding activity in human B-lineage lymphoid cells

We next examined the effects of the BTK kinase inhibitor LFM-A13 on serine phosphorylation of native IK in human B-lymphoblastoid cell line BCL1. Engagement of the CD19 receptor results in activation of BTK as well as the related kinase TEC in human B-lineage lymphoid cells [27] and thereby provides a model for studying BTK-IK interactions. Western blot analysis of IK immune complexes from BCL1 cells with an anti-phosphoserine antibody showed increased serine phosphorylation after stimulation with a CD19-receptor specific monoclonal antibody homoconjugate (CD19×CD19). BTK-inhibitor LFM-A13 blocked this response to CD19 engagement and prevented CD19×CD19-induced serine phosphorylation of IK (**Figure S4A in File S1**). The negative effect of LFM-A13 on this activation-induced phosphorylation indicated that BTK was directly responsible for serine phosphorylation of native IK after CD19 receptor engagement. We also examined the effects of LFM-A13 on CD19xCD19-induced IK activation in BCL1 cells using EMSA. Stimulation of BCL1 cells with CD19×CD19 homoconjugate for 2 hours increased the sequence-specific DNA binding activity of native IK (**Figure S4B in File S1**, Lane 2 vs Lane 7). LFM-A13 treatment prevented CD19×CD19-induced IK activation in a concentration-dependent fashion (**Figure S4B in File S1**, Lanes 3–6). The specificity of the binding was confirmed by demonstrating that the mobility shifts caused by binding of native IK in NE to the radiolabeled IK-BS1 probe are abrogated via cold competition using excess unlabeled IKBS1 (Lane 8). No mobility shifts were observed with the control oligonucleotide probe IK-BS5 that has a single base pair (G>A) substitution at position 3 within the core consensus and does not bind IK (Lanes 9 and 10).

### BTK phosphorylates Ikaros at unique phosphorylation sites serine 214 and serine 215

**Figure 4:**
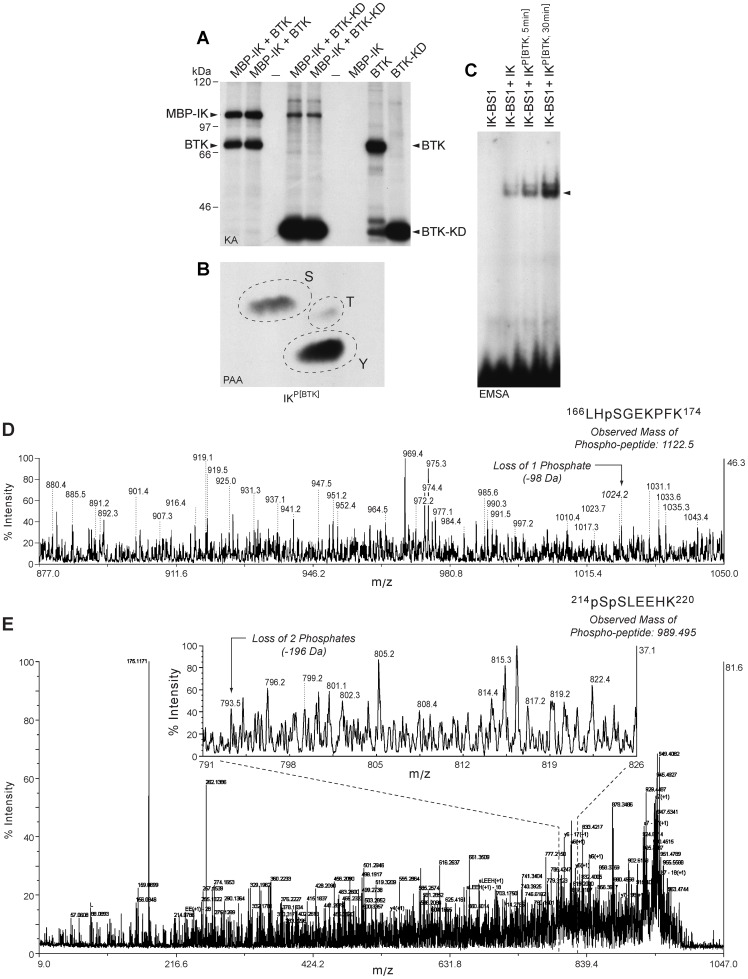
Phosphorylation and Activation of Recombinant Ikaros by Recombinant BTK and BTK-KD. [**A**] Recombinant full-length BTK as well as BTK-KD showed autophosphorylation and it also phosphorylated MBP-tagged recombinant IK1 in hot kinase assays in the presence of [gamma-^32^P]ATP. Depicted is the autoradiograph of the kinase reaction products. The positions of the autophosphorylated BTK kinase band and BTK kinase domain band as well as the phosphorylated recombinant IK band are indicated with arrows. [**B**] Phospho amino acid analysis of the BTK phosphorylated MBP-IK1 band showed phosphorylation on tyrosine and serine. The ^32^P-labeled MBP-IK1 band in [A] was isolated and subjected to PAA. The positions of ninhydrin-stained phosphoamino acid standards (phosphoserine [S], phosphothreonine [T], and phosphotyrosine [Y]) are indicated with circles. [**C**] EMSA's were performed with a ^32^P-labeled double-stranded IK-BS1 oligonucleotide probe containing a high-affinity IK binding site (1 ng/sample, 100,000 cpm) and purified MBP-tagged recombinant IK1 protein (200 ng/sample) that has or has not been phosphorylated by recombinant BTK as in panel A. Lane 1, IK-BS1 probe only. Lane 2, unphosphorylated IK1 (200 ng) was mixed with 1 ng radiolabeled IK-BS1. Lane 3/Lane 4, BTK-phosphorylated MBP-IK1 (MBP-IK1^Phos(BTK)^) was mixed after a 5 min vs. 30 min kinase assay with 1 ng radiolabeled IK-BS1. MBP-IK1^Phos(BTK)^ exhibited augmented binding to IK-BS1 when compared to MBP-IK1. [D&E] Mass spectrographs of phosphopeptides from BTK-phosphorylated recombinant IK1 protein. A MALDI-TOF/TOF mass spectrometry analysis was performed on trypsin-digested recombinant IK1 after an in vitro kinase reaction with purified recombinant BTK. Supel-Tips (Sigma-Aldrich) were used for phosphopeptide enrichment. Tryptic peptides were desalted and concentrated using the Millipore C18 reverse phase Zip-Tips column, eluted in 0.5 μL of matrix solution and spotted on the MALDI plate. MALDI-TOF MS was performed on an AB Sciex Proteomics Analyzer. MS spectra were acquired in reflection positive ion mode, averaging 4000 laser shots/per spectrum. The masses of the peptides after neutral loss 1 (−98 kDa) or 2 (−196 kDa) are indicated with arrows. [D] CID spectrum shows neutral loss of 1 phosphate indicating that S168 serves as a target phosphorylation site for BTK. [E] CID spectrum reveals neutral loss of 2 phosphates indicating that S214 and S215 residues of IK serve as unique target phosphorylation sites for BTK.

We next performed kinase assays to determine if purified recombinant BTK and its kinase domain (KD) can phosphorylate purified MBP-tagged recombinant IK1 in vitro. Both full-length BTK and BTK-KD phosphorylated recombinant IK1 (**Figure 4A**). Phosphoaminoacid analysis of the BTK-phosphorylated IK1 protein in hot kinase assays showed that BTK phosphorylates IK1 on both tyrosine and serine residues (**Figure 4B**). Notably, BTK-induced phosphorylation of recombinant IK1 augmented its sequence-specific DNA binding activity in a cell-free EMSA platform in the absence of other proteins (**Figure 4C**).

A matrix-assisted laser desorption/ionization–time-of-flight (MALDI-TOF)/TOF mass spectrometry analysis was performed on trypsin-digested recombinant IK1 after an in vitro kinase reaction with purified recombinant BTK. After TiO_2_ enrichment, 2 precursors at mass 989.49 Da and mass 1122.5 Da corresponding to phosphorylated peptides ^214^pSpSLEEHK^220^ and ^166^LHpSGEKPFK^174^, respectively, were identified with subsequent CID spectra revealing peaks of the corresponding mass losses of 196 Da and 98 Da, which indicates that Ser^168^ (S168) (**Figure 4D**), Ser^214^ (S214) and Ser^215^ (S215) are BTK phosphorylation sites (**Figure 4E**). Residues S214 and S215 are novel phosphorylation sites, and the peptide containing these two sites corresponds to a consensus sequence encoded by Exon 5 in the vicinity of ZF4 and found in IK from mouse (NM_001025597, human (NM_006060.4), and chicken (NM_205088). Although BTK also phosphorylated S168 residue of IK1 in a segment between ZF2 and ZF3 that is encoded by Exon 4, phosphorylation of this site is previously reported to have no functional significance [28]. No tyrosine phosphorylation sites could be identified, likely due to inherent technical difficulties in identifying tyrosine phosphorylation sites using (MALDI-TOF)/TOF mass spectrometry analysis.

### Site-directed mutagenesis of Ikaros at unique BTK phosphorylation sites alters its DNA binding activity and subcellular localization

**Figure 5:**
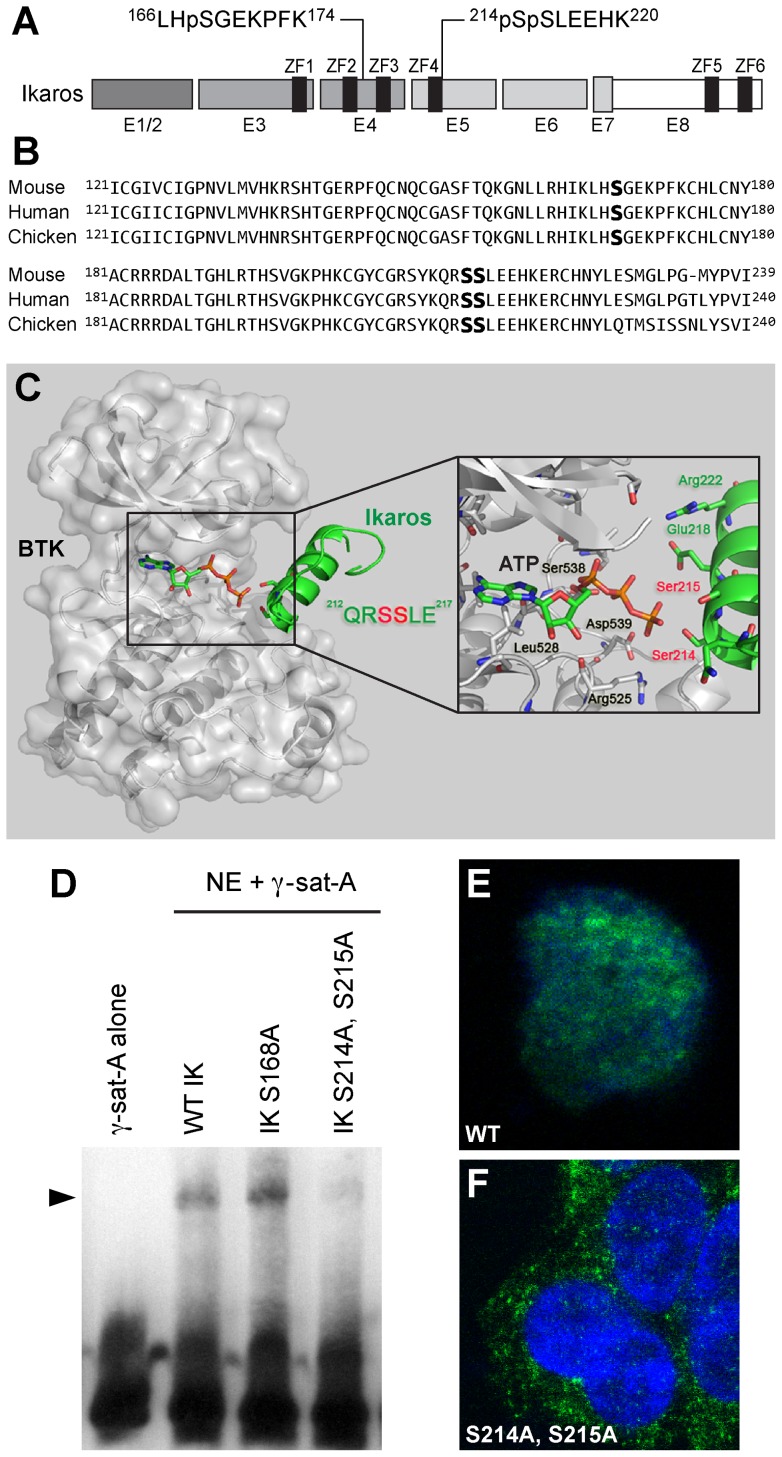
BTK phosphorylation sites of Ikaros. [**A**] We identified Ser^214^ (S214) and Ser^215^ (S215) as two unique BTK phosphorylation sites within the mouse IK peptide ^214^SSLEEHK^220^ corresponding to a consensus sequence encoded by Exon 5 and found in IK from mouse (NM_001025597, human (NM_006060.4), and chicken (NM_205088). [**B**] Alignment of mouse, human, and chicken IK protein segments containing the identified BTK phosphorylation sites. [**C**] Schematic diagram of BTK-IK (203–224) complex. BTK is shown as a molecular surface colored in gray, and IK is shown as secondary structure colored in green. Key residues in catalytic site of BTK, ATP and substrate residues (Ser214 and Ser215) in the peptide of IK are shown as a stick model. The model was built by Modeler [1] and shown with Pymol. [**D**] Loss of IK DNA binding activity by BTK-resistance mutations. EMSAs were performed on nuclear extracts (NE) from Hek293T cells transfected with expression vectors for wildtype (WT) or mutant IK proteins using the Thermo Scientific LightShift Chemiluminescent EMSA Kit and biotin-labeled DNA probe gamma-satellite A. IK activity was measured by the electrophoretic mobility shifts of the biotin-labeled probe, representing IK-containing nuclear complexes (indicated with arrow head). The biotin-labeled DNA was detected using streptavidin-horseradish peroxidase conjugate and a chemiluminescent substrate. The membrane was exposed to X-ray film and developed with a film processor. [E] Confocal two-color merge image of a representative Hek293T cell expressing FLAG-tagged wildtype IK protein (green) in the DAPI-stained (blue) nucleus. (System Magnification: 630×). [F] Confocal two-color merge image of representative Hek293T cells expressing FLAG-tagged mutant IK protein with alanine substitutions at BTK phosphorylation sites S214 and S215 (green) in the cytoplasm outside the DAPI-stained nucleus. (System Magnification: 630×). To detect the FLAG-tagged wildtype (in E) and BTK-phosphorylation site mutant IK proteins (in F), cells were stained with a monoclonal mouse anti-DDK antibody and a secondary goat anti-mouse antibody conjugated with Alexa Fluor 488. Fluorescent cells were imaged using the PerkinElmer Ultraviewer Confocal Dual Spinning Disc Scanner (Shelton, CT).

The BTK phosphorylation sites S214 and S215 are very close to the N-terminal ZF4 of the IK DNA binding domain (**Figure 5A&B**). We constructed a structural model of BTK in complex with the IK peptide (residues 203–224) to investigate the mechanism of BTK-induced serine phosphorylation of IK on S214 and S215 residues at an atomic level (**Figure 5C**). This model shows that the alpha-helix of the target IK peptide would readily bind to BTK catalytic site with a compact conformation due to its narrow and deep shape, leading to an microenvironment of transphosphorylation composed of ATP, S214 and S215 residues of human IK catalytic residues (e.g. D539) in BTK, and other potential key residues in IK (e.g. E218 and R222). According to this model, the terminal phosphate of ATP can easily be transferred by BTK to either S214 or S215 of IK (**Figure 5C**). The contribution of BTK-induced phosphorylation of S168, S214, and S215 residues to IK function was determined by performing site-directed mutagenesis to replace these amino acids with alanine for eliminating the effects of BTK-induced phosphorylation. IK has been shown to bind to repetitive sequences within PC-HC that contain consensus IK binding sites, and its localization to the PC-HC in the nucleus is directly related to its ability to bind to these sequences [28]. We performed EMSAs to directly examine the effect of mutations at BTK phosphorylation sites on the binding of IK to the biotin labeled gamma-satellite A probe [2] derived from the centromeric gamma-satellite repeat sequences [29]. The gamma-satellite A DNA probe contains two consensus IK binding sites in close proximity to each other and is a target for high affinity binding of wildtype IK and the binding affinity to this probe shows an excellent correlation with the homing of IK to PC-HC (28). Notably, alanine substitution of the BTK phosphorylation sites S214 and S215 (but not S168) abolished the ability of native IK to bind to the gamma-satellite A probe (**Figure 5D**). Further, IK with alanine substitution of the BTK phosphorylation sites S214 and S215 showed a strictly cytoplasmic localization (**Figure 5E, 5F**). These results confirm and extend the results of RNAi experiments and provide direct genetic evidence that BTK-induced serine phosphorylation of IK at S214 and S215 affects its DNA binding activity. By comparison, phenylalanine substitutions of all 4 tyrosine residues (Y292, Y409, Y493 and Y499) predicted to be the most likely of the 16 tyrosines in IK to serve as phosphorylation sites based on their NetPhos prediction scores did not affect the binding of IK to the gamma-satellite A or IK-BS1 DNA probes [**13**].

## Discussion

IK is a zinc finger-containing DNA-binding protein that plays a pivotal role in immune homeostasis through transcriptional regulation of the earliest stages of lymphocyte ontogeny by both (a) gene transcriptional activation via efficient transcription initiation and elongation as well as (b) gene repression [10]–[12], [26]. IK also exhibits a tumor suppressor function in lymphocyte precursors, which has been attributed in part to its ability to repress expression of oncogenic genes via chromatin remodeling in association with the SWI/SNF remodeling complex and recruitment of potentially oncogenic proliferation-promoting genes to pericentromeric heterochromatin (PC-HC) [10]–[12], [26], [30]. Impaired DNA binding activity of IK has been associated with a release of NuRD from IK target genes to cause both a maturational arrest in lymphocyte ontogeny and an “illegitimate” activation of a network of genes that promote leukemogenesis [30]. Functional deficiency of IK has been implicated in the pathogenesis of acute lymphoblastic leukemia, the most common form of childhood cancer [26], [31], [32]. Therefore, a stringent regulation of IK activity is considered of paramount importance.

Currently, our knowledge regarding the upstream regulators of IK function is very limited. Phosphorylation of IK by CK2 inhibits its many functions and promotes its degradation via the ubiquitin/proteosome pathway [17], [26]. Conversely, dephosphorylation of IK by protein phosphatase 1 (PP1) is critical for its ability to bind to target DNA sequences, localize to PC-HC in the nucleus, and exert its regulatory functions [17]. Besides the CK-PP1 molecular complex, SYK has also been identified as a regulator of IK function [13].

BTK is a member of the SRC-related TEC family of cytoplasmic PTK [1]–[4]. BTK is a physiologically important kinase that serves as a key regulator of multiple biochemical signal transduction events and biologic responses in B-lineage lymphoid cells throughout B-cell ontogeny [1]–[4]. Our results presented herein now provide the first genetic and biochemical evidence for a previously unknown serine kinase function of BTK as a partner of IK that physically associates with IK and phosphorylates it at unique phosphorylation sites thereby augmenting its nuclear localization and sequence-specific DNA binding activity. We further show that BTK regulates the expression levels of validated IK target genes in human cells. BTK induced phosphorylation is mandatory for optimal nuclear localization and transcription factor function of IK in human cells. Functional analysis of mutant IK proteins and evaluation of the effects of BTK siRNA on IK function demonstrated that BTK-induced serine phosphorylation is mandatory for the nuclear localization and DNA binding of both wildtype IK and IK with alanine or aspartate mutations at CK2-phosphorylation sites. As both BTK and IK reside in the cytoplasm as well as the nucleus, BTK-induced activating phosphorylation of IK could occur in the cytoplasm or nucleus. The documented effects of BTK-induced phosphorylation of IK are reminiscent of the effects of SYK-induced IK phosphorylation [13], although the phosphorylation sites are not the same. The presented results corroborate the growing evidence that multiple counter-regulatory mechanisms exist and operate to regulate the function of IK, thereby ensuring the orderly development and differentiation of B-cell precursors. BTK phosphorylated IK on both serine and tyrosine residues. Although the identity of the tyrosine phosphorylation sites could not be determined in the present study, phenylalanine substitutions of Y292, Y409, Y493 and Y499 predicted to be the most likely of the 16 tyrosine residues in IK to serve as putative tyrosine phosphorylation sites based on their NetPhos prediction scores did not affect the DNA binding activity of IK to the gamma-satellite A [13].

The relative contributions of SYK and BTK to the transcription factor function of IK will be the subject of our future research efforts. SYK has been shown to cooperate with BTK via the B cell-specific adapter molecule BLNK/SLP-65 in phosphorylation and activation of multiple intracellular effector molecules in the context of B-cell antigen receptor signaling [14]. Furthermore, SYK directly activates BTK by phosphorylating the Y551 residue in the activation loop of the BTK catalytic domain [14]. Notably, the SYK phosphorylation site mutants of IK without any mutation at the BTK phosphorylation sites [13] as well as the BTK phosphorylation site mutants of IK without any mutation at the SYK phosphorylation sites (present study) exhibited aberrant subcellular localization and defective DNA binding. Therefore, we propose that cooperation between SYK and BTK may also be important for the nuclear localization and optimal function of IK.

The 3-D structure of IK has not been resolved and the exact roles of the 6 C_2_H_2_ ZFs of IK in its interactions with DNA and their relative contributions to its DNA binding affinity remain unknown [33]. The BTK phosphorylation sites S214 and S215 are very close to the N-terminal ZF4 of the IK DNA binding domain. BTK mediated phosphorylation of the IK protein may induce a conformational change that affects the accessibility and DNA binding affinity of ZF4. Furthermore, this phosphorylation adjacent to ZF4 may also affect the overall protein conformation of IK and the DNA binding affinity of all of its N-terminal ZF. The future elucidation of the structural basis of IK activation by BTK-induced serine phosphorylation will require a 3–D structure determination of IK at atomic resolution before and after phosphorylation using X-ray crystallography or nuclear magnetic resonance (NMR) spectroscopy.

## Supporting Information

File S1 **Figure S1, Expression Levels of Lymphoid-priming Genes in Primary Lymphocyte Precursors from B-lineage ALL Patients in Relationship to BTK Transcript Levels.** [A] Gene Pattern (http://www.broadinstitute.org/cancer/software/genepattern/) was used to extract expression values for the 20 Lymphoid-priming genes in the combined data set from 5 studies with a total of 1104 primary leukemia samples of human lymphocyte precursors for further analysis (18 genes/ 27 transcripts were represented on the Human gene chips). Expression values expressed as Standard Deviation units calculated from 1104 samples were compiled for the 5 studies and rank ordered according to the mean expression of 3 highly correlated the *BTK* transcripts. For each study, the standard deviation values were calculated from the study mean for all the patients. We focused our analysis on human lymphocyte precursors from 884 B-lineage ALL patients. The datasets were combined to test for consistent differences in the Z-scores for high *BTK* (>0.5 SD units; N = 369 samples) and low *BTK* (<−0.5 SD units; N = 125 samples) groups. These samples were also rank ordered according to *IKZF1* expression level (205038_at, 205039_s_at, 216901_s_at, 227344_at and 227346_at; 3 of these were common in all Affymetrix platforms (205038_at, 205039_s_at, 216901_s_at)). A one-way agglomerative hierarchical clustering technique was used to organize expression patterns using the average distance linkage method such that genes (rows) having similar expression across patients were grouped together (average distance metric). The heat map depicts expression values represented by standard deviation units above (red) and below the mean (green). Dendrograms were drawn to illustrate similar gene-expression profiles from joining pairs of closely related gene expression profiles, whereby genes joined by short branch lengths showed most similarity in expression profile across patients. [B] Standardized expression values (SD units) for *BTK* probeset (205504_at) compiled from the 5 studies comprising of 1104 primary leukemia specimens from pediatric acute lymphoblastic leukemia (ALL) patients (GSE3912, N = 113; GSE18497, N = 82; GSE4698, N = 60; GSE7440, N = 99; GSE13159, N = 750) were examined for correlation with the expression of 3 *Ikaros/IKZF1* probesets common in all 5 studies (205038_at, 205039_s_at, 216901_s_at). Highly significant correlations were observed for *IKZF1* probesets plotted against the *BTK* probeset: 205038_at (**B.1**; Correlation Coefficient  = 0.27, T-value  = 9.24, P<0.0001), 205039_s_at (**B.2**; Correlation Coefficient  = 0.19, T-value  = 6.47, P<0.0001) and 216901_s_at (**B.3**; Correlation Coefficient  = 0.19, T-value  = 6.37, P<0.0001). Line of best fit and the associated 95% confidence intervals for the fit are shown in the shaded area. [C] T-tests were performed for the combined Standard Deviation units from the 5 datasets (2-sample, Unequal variance correction, p-values<0.05 deemed significant) to reveal 15 transcripts representing 12 lymphoid priming genes significantly upregulated in specimens with both high *BTK* and high *IKZF1* expression. **Figure S2, Expression Levels of Validated Ikaros Target Genes in Primary Lymphocyte Precursors from B-lineage ALL Patients in Relationship to** ***BTK*** **Transcript Levels.** Gene Pattern (http://www.broadinstitute.org/cancer/software/genepattern/) was used for further analysis of expression values for previously published and validated IK target genes [13] in the combined data set from 5 studies with a total of 884 primary leukemia samples of human lymphocyte precursors from B-lineage ALL patients. For each study, the standard deviation values were calculated from the study mean for all the patients. A one-way agglomerative hierarchical clustering technique was used to organize gene expression patterns using the average distance linkage method such that genes (rows) having similar expression across patients were grouped together (average distance metric). The heat map depicts expression values represented by standard deviation units above (red) and below the mean (green). Dendrograms were drawn to illustrate similar gene-expression profiles by joining pairs of closely related gene expression profiles, whereby genes joined by short branch lengths showed most similarity in expression profile across patients. Samples were assigned to the “high *BTK* expression” group if their expression level was >0.5 standard deviation unit higher than the mean expression level (N = 369) and to the “low *BTK* expression” group if their expression level was >0.5 standard deviation unit lower than the mean expression level (N = 125) resulted in identification of 25 IK target genes upregulated in high *BTK* expression group. T-tests were performed using standardized expression values combined from 5 datasets (2-sample, Unequal variance correction, p-values<0.05 deemed significant) revealing an intersect of 34 transcripts representing 24 genes that were significantly up-regulated in both high *BTK* and high *IKZF1* expression groups (29 genes were up regulated in high *IKZF1* samples, of which 5 were not upregulated in high *BTK* samples versus 24 upregulated in both high *IKZF1* and high *BTK* samples, Fisher's Exact Test, 1-Tailed, P <0.0001). Hierarchical cluster analysis identified a set of genes highly co-regulated with *BTK* expression: *LAMC1* (2 transcripts; 0.68 and 0.97 SD Units, P = 9.2×10^−16^ and 7.3×10^−33^ respectively; *PTK2* (2 transcripts; 0.42 and 0.46 SD Units, P = 4.5×10^−6^ and 2.7×10^−6^ respectively); *TSPAN13* (0.63 SD units, P = 2.3×10^−17^); *PREP* (2 transcripts; 0.82 and 0.89 SD Units, P = 2.3×10^−18^ and 7.1×10^−22^ respectively); and *SERPINI1* (0.84 SD units, P = 1.7×10^−27^) were the most significantly up-regulated 5 genes with the most significant effect sizes in 369 patient samples with high BTK expression. **Figure S3, Co-localization and Physical Interactions of Native Ikaros and BTK Proteins in Human Cells.** [A] Nuclear co-localization of Native IK and BTK. RAJI and DAUDI cells were fixed and stained with polyclonal rabbit anti-IK1 (primary Ab)/ Alexa Fluor 568 F(ab')_2_ fragment of goat anti-rabbit IgG (secondary Ab) (red) and mouse anti-BTK MoAb (primary Ab)/ Alexa Fluor 488 goat anti-mouse IgG (secondary Ab) (green) antibodies. Nuclei were stained with blue fluorescent dye 4′,6-diamidino-2-phenylindole (DAPI). MERGE panels depict the merge three-color confocal image showing co-localization of IK1 and BTK in DAPI-stained nucleus as magenta immunofluorescent foci (System magnification: 315×). [B & C] Co-immunoprecipitation of Native IK and BTK. [B] depicts the results of the BTK and IK Western blot analysis of the IK immune complexes immunoprecipitated (IP) from RAJI and DAUDI cells. [C] depicts the results of the IK and BTK Western blot analysis of the BTK immune complexes from the same cells. **Figure S4, Effects of BTK Inhibition on CD19-Mediated Activation of IK in Human B-lineage Lymphoid Cells.** [A] Anti-phosphoserine (A.1) and Anti-IK (A.2) Western blot analysis of IK immune complexes from BCL1 cells before and after stimulation with an anti-CD19 monoclonal antibody homoconjugate (CD19xCD19) (1 µg/mL) for 15 min and 30 min. A.1: CD19 engagement caused increased serine phosphorylation of IK, which was abrogated by the BTK inhibitor LFM-A13 (50 µM). A.2: LFM-A13 treatment did not affect the IK protein level. [B] EMSA was performed using nuclear extracts (NE) from BCL-1 cells with and without a 2-hour stimulation with an anti-CD19 monoclonal antibody homoconjugate (CD19xCD19) (1 µg/mL) using the radiolabeled *IK-BS1* oligonucleotide probe containing a high-affinity IK1 binding site. CD19 engagement caused increased the sequence-specific DNA binding activity of native IK (Lane 7 vs. Lane 2) and this response was blocked by the BTK inhibitor LFM-A13 in a concentration-dependent fashion (Lanes 3–6). The specificity of the binding was confirmed by demonstrating that the mobility shifts caused by binding of native IK in NE to the radiolabeled IK-BS1 probe are abrogated via homologous competition using 60-fold molar excess unlabeled IKBS1 probe (Lane 8). Lanes 9&10: No mobility shifts were observed with the control oligonucleotide probe IK-BS5 that has a single base pair (G>A) substitution at position 3 within the core consensus and does not bind IK.
(PDF)
