# Supplementary material for: Regulatory Phosphorylation of Ikaros by Bruton's Tyrosine Kinase
Source: PLoS One. 2013 Aug 19;8(8):e71302. doi: 10.1371/journal.pone.0071302 (PMC3747153; doi:10.1371/journal.pone.0071302)
Supplement: File S1 — Figure S1, Expression Levels of Lymphoid-priming Genes in Primary Lymphocyte Precursors from B-lineage ALL Patients in Relationship to BTK Transcript Levels. [A] Gene Pattern (http://www.broadinstitute.org/cancer/software/genepattern/) was used to extract expression values for the 20 Lymphoid-priming genes in the combined data set from 5 studies with a total of 1104 primary leukemia samples of human lymphocyte precursors for further analysis (18 genes/ 27 transcripts were represented on the Human gene chips). Expression values expressed as Standard Deviation units calculated from 1104 samples were compiled for the 5 studies and rank ordered according to the mean expression of 3 highly correlated the BTK transcripts. For each study, the standard deviation values were calculated from the study mean for all the patients. We focused our analysis on human lymphocyte precursors from 884 B-lineage ALL patients. The datasets were combined to test for consistent differences in the Z-scores for high BTK (>0.5 SD units; N = 369 samples) and low BTK (<−0.5 SD units; N = 125 samples) groups. These samples were also rank ordered according to IKZF1 expression level (205038_at, 205039_s_at, 216901_s_at, 227344_at and 227346_at; 3 of these were common in all Affymetrix platforms (205038_at, 205039_s_at, 216901_s_at)). A one-way agglomerative hierarchical clustering technique was used to organize expression patterns using the average distance linkage method such that genes (rows) having similar expression across patients were grouped together (average distance metric). The heat map depicts expression values represented by standard deviation units above (red) and below the mean (green). Dendrograms were drawn to illustrate similar gene-expression profiles from joining pairs of closely related gene expression profiles, whereby genes joined by short branch lengths showed most similarity in expression profile across patients. [B] Standardized expression values (SD units) for BTK probeset [file pone.0071302.s001.pdf]

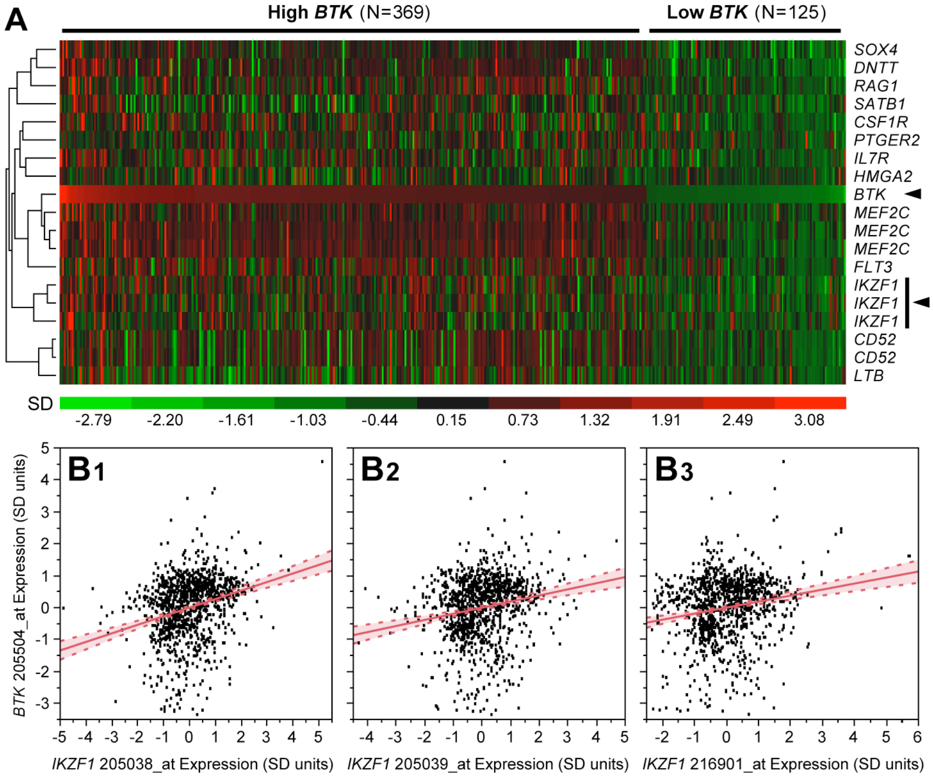

C

Affy ID	Gene symbol	StdDev [High *BTK* (N = 369) – Low *BTK* (N = 125)]	T-test P-value	StdDev [High *Ikaros* (N = 258) – Low *Ikaros* (N = 247)]	T-test P-value
209200_at	*MEF2C*	1.04	9.7×10^{-43}	0.89	2.3×10^{-38}
210487_at	*DNTT*	0.93	5.6×10^{-24}	0.47	7.6×10^{-08}
209199_s_at	*MEF2C*	0.83	2.7×10^{-23}	0.69	1.6×10^{-31}
207968_s_at	*MEF2C*	0.71	4.3×10^{-19}	0.62	2.4×10^{-17}
206591_at	*RAG1*	0.72	2.5×10^{-18}	0.72	8.2×10^{-18}
205798_at	*IL7R*	0.65	1.9×10^{-14}	0.56	1.6×10^{-09}
206674_at	*FLT3*	0.62	1.6×10^{-13}	0.47	4.2×10^{-10}
201417_at	*SOX4*	0.74	1.8×10^{-13}	0.40	3.6×10^{-06}
34210_at	*CD52*	0.55	3.0×10^{-12}	0.22	9.7×10^{-03}
203104_at	*CSF1R*	0.68	2.5×10^{-11}	0.39	1.9×10^{-05}
204661_at	*CD52*	0.52	1.1×10^{-10}	0.23	6.8×10^{-03}
203408_s_at	*SATB1*	0.32	4.9×10^{-04}	0.59	4.7×10^{-12}
206631_at	*PTGER2*	0.32	1.2×10^{-03}	0.54	2.5×10^{-09}
207339_s_at	*LTB*	0.28	1.3×10^{-03}	0.41	9.6×10^{-06}
208025_s_at	*HMGA2*	0.22	1.1×10^{-02}	0.36	4.4×10^{-06}
205038_at	*IKZF1*	0.83	1.1×10^{-17}	2.26	8.8×10^{-152}
205039_s_at	*IKZF1*	0.60	4.5×10^{-10}	2.08	5.4×10^{-123}
216901_s_at	*IKZF1*	0.42	2.5×10^{-06}	1.69	7.5×10^{-76}
205504_at	*BTK*	1.70	4.7×10^{-177}	0.62	4.8×10^{-23}

Figure S1. Expression Levels of Lymphoid-priming Genes in Primary Lymphocyte Precursors from B-lineage ALL Patients in Relationship to BTK Transcript Levels.

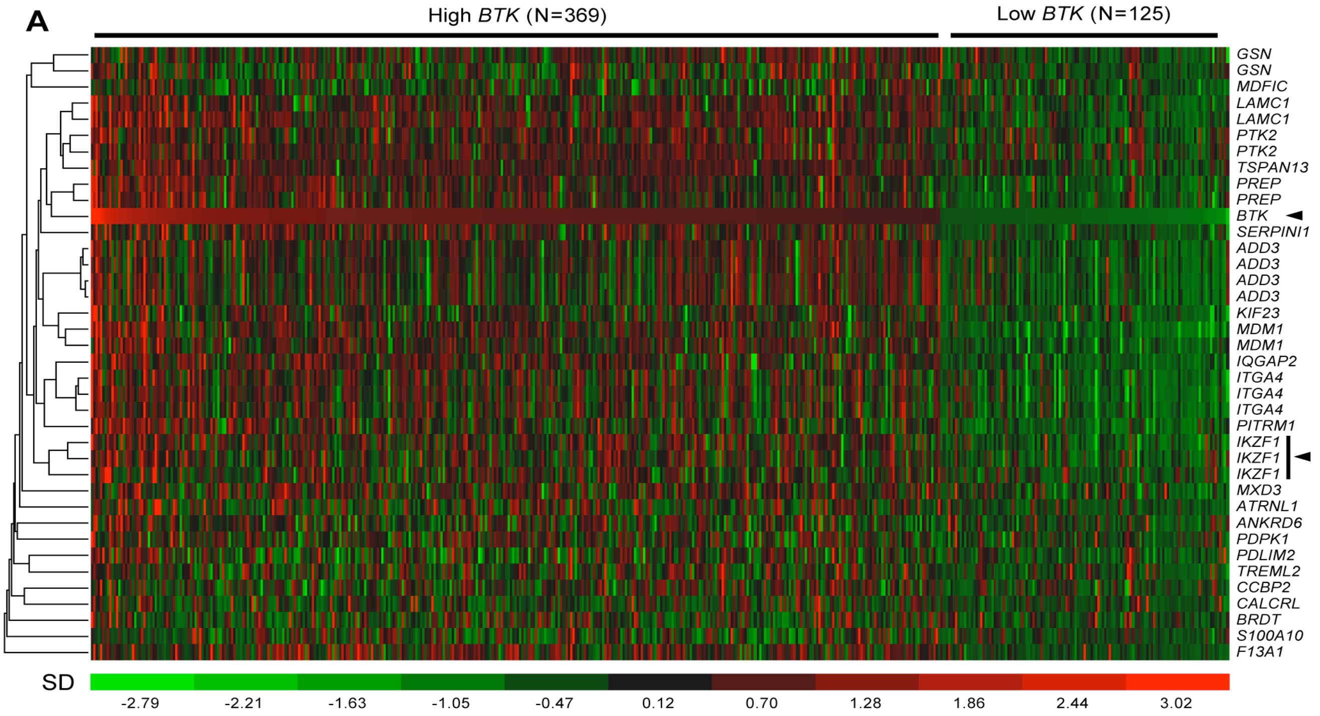

B

Affy ID	Gene symbol	StdDev [High *BTK* (N = 369) – Low *BTK* (N = 125)]	T-test P-value	StdDev [High *Ikaros* (N = 258) – Low *Ikaros* (N = 247)]	T-test P-value
213761_at	*MDM1*	1.20	3.5×10^{-40}	0.89	1.7×10^{-25}
200771_at	*LAMC1*	0.97	7.3×10^{-33}	0.78	8.9×10^{-29}
203474_at	*IQGAP2*	0.91	6.6×10^{-31}	0.65	3.9×10^{-15}
205352_at	*SERPINI1*	0.84	1.7×10^{-27}	0.61	3.5×10^{-12}
220397_at	*MDM1*	0.93	2.2×10^{-27}	0.82	2.2×10^{-22}
205273_s_at	*PITRM1*	0.85	1.3×10^{-23}	0.54	2.6×10^{-10}
204117_at	*PREP*	0.89	7.1×10^{-22}	0.66	4.3×10^{-12}
205884_at	*ITGA4*	0.88	2.7×10^{-20}	0.96	8.0×10^{-27}
201034_at	*ADD3*	0.75	6.3×10^{-20}	0.67	8.2×10^{-16}
205885_s_at	*ITGA4*	0.82	2.1×10^{-19}	0.84	3.9×10^{-22}
213416_at	*ITGA4*	0.90	2.3×10^{-19}	0.87	3.8×10^{-24}
37950_at	*PREP*	0.82	2.3×10^{-18}	0.58	4.4×10^{-11}
201752_s_at	*ADD3*	0.71	2.2×10^{-17}	0.59	3.1×10^{-12}
217979_at	*TSPAN13*	0.63	2.3×10^{-17}	0.46	6.2×10^{-16}
205882_x_at	*ADD3*	0.71	1.3×10^{-16}	0.58	1.1×10^{-11}
200770_s_at	*LAMC1*	0.68	9.2×10^{-16}	0.33	7.4×10^{-06}
203305_at	*F13A1*	0.71	1.4×10^{-15}	0.39	5.4×10^{-06}
201753_s_at	*ADD3*	0.68	9.2×10^{-15}	0.59	3.5×10^{-13}
204709_s_at	*KIF23*	0.59	1.5×10^{-11}	0.48	4.3×10^{-09}
213745_at	*ATRNL1*	0.48	2.8×10^{-09}	0.32	1.1×10^{-03}
200696_s_at	*GSN*	0.48	9.0×10^{-08}	0.41	7.7×10^{-08}
207821_s_at	*PTK2*	0.46	2.8×10^{-06}	0.43	6.2×10^{-08}
221250_s_at	*MXD3*	0.51	3.0×10^{-06}	0.24	7.6×10^{-03}
208820_at	*PTK2*	0.42	4.5×10^{-06}	0.26	6.6×10^{-05}
217599_s_at	*MDFIC*	0.27	1.7×10^{-03}	0.56	1.0×10^{-10}
219748_at	*TREML2*	0.28	2.5×10^{-03}	0.42	4.4×10^{-06}
221244_s_at	*PDPK1*	0.29	2.5×10^{-03}	0.41	6.9×10^{-06}
214040_s_at	*GSN*	0.25	6.4×10^{-03}	0.41	8.8×10^{-06}
219165_at	*PDLIM2*	0.25	8.7×10^{-03}	0.31	4.9×10^{-04}
204672_s_at	*ANKRD6*	0.25	1.1×10^{-02}	0.39	9.1×10^{-06}
206887_at	*CCBP2*	0.23	1.2×10^{-02}	0.31	7.0×10^{-04}
206787_at	*BRDT*	0.21	2.5×10^{-02}	0.29	2.2×10^{-03}
200872_at	*S100A10*	0.16	4.4×10^{-02}	0.23	8.5×10^{-03}
210815_s_at	*CALCRL*	0.18	4.9×10^{-02}	0.27	3.0×10^{-03}
205038_at	*IKZF1*	0.83	1.1×10^{-17}	2.26	8.8×10^{-152}
205039_s_at	*IKZF1*	0.60	4.5×10^{-10}	2.08	5.4×10^{-123}
216901_s_at	*IKZF1*	0.42	2.5×10^{-06}	1.69	7.5×10^{-76}
205504_at	*BTK*	1.70	4.7×10^{-177}	0.62	4.8×10^{-23}

Figure S2. Expression Levels of Validated Ikaros Target Genes in Primary Lymphocyte Precursors from B-lineage ALL Patients in Relationship to *BTK* Transcript Levels.

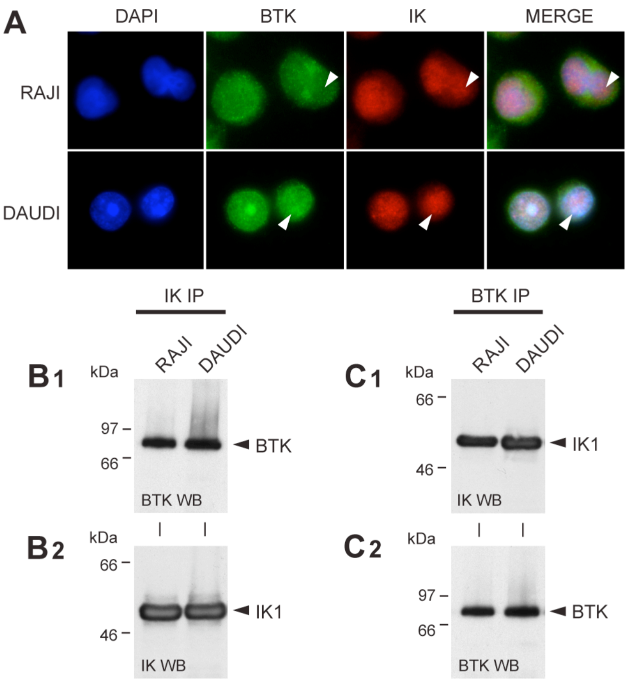

Figure S3. **Co-localization and Physical Interactions of Native Ikaros and BTK Proteins in Human Cells.**

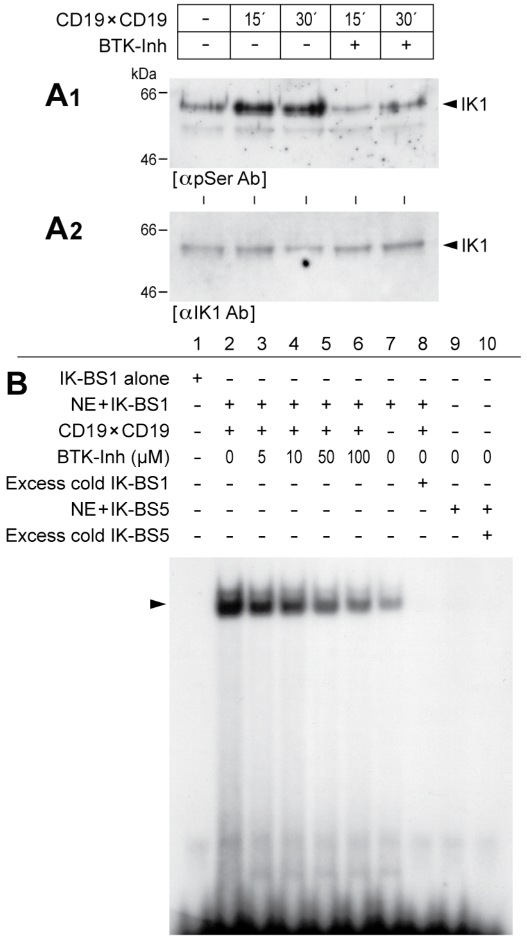

Figure S4. Effects of BTK Inhibition on CD19-Mediated Activation of IK in Human B-lineage Lymphoid Cells.
